# Keratin utilizes H_2_S to promote HMGB1 sulfhydration for inflammation resolution

**DOI:** 10.1016/j.redox.2026.104326

**Published:** 2026-07-31

**Authors:** Xiaozheng Huang, Wanglin Bao, Shengming Tang, Miao Li, Yuqing Sun, Hui Ge, Yuxin Zhao, Hao Yuan, Xinyu Pan, Wei Lin, Yuanqing Wei, Shuying Han, Wenxing Wu, Rui Liu, Jin-ao Duan

**Affiliations:** a State Key Laboratory of Technologies for Chinese Medicine Pharmaceutical Process Control and Intelligent Manufacture (Nanjing University of Chinese Medicine), Nanjing, Jiangsu, 211112, China; b Jiangsu Collaborative Innovation Center of Chinese Medicinal Resources Industrialization, National and Local Collaborative Engineering Center of Chinese Medicinal Resources Industrialization and Formulae Innovative Medicine, National Administration of Traditional Chinese Medicine Key Laboratory for Chinese Medicine Resources Recycling Utilization, Nanjing University of Chinese Medicine, Nanjing, 210023, China; c Jiangsu Key Laboratory of Research and Development in Marine Bio-resource Pharmaceutics, Nanjing University of Chinese Medicine, Nanjing, 210023, China; d Animal-Derived Chinese Medicine and Functional Peptides International Collaboration Joint Laboratory, Nanjing, 210023, China; e School of Pharmacy, Nanjing University of Chinese Medicine, Nanjing, 210023, China; f State Key Laboratory for Quality Ensurance and Sustainable Use of Dao-di Herbs, Beijing, 100700, China; g Jinhua Academy of Zhejiang Chinese Medical University, Jinhua, Zhejiang, 321015, China

**Keywords:** Keratin, Oxidative-inflammatory stress, H_2_S homeostasis, Protein redox state, Sulfhydration of HMGB1

## Abstract

Despite the established importance of hydrogen sulfide (H_2_S) in redox biology, strategies to modulate its endogenous levels remain largely unexplored. Here, we show that a keratin fraction (KF) obtained from natural sources modulates H_2_S homeostasis and protein redox state through sulfhydration. Chemoproteomic analysis identified keratin 2 (K2; UniProtKB P25691, keratin, type II microfibrillar, component 5) as the most hyperreactive keratin toward sulfhydration. Oral administration of K2 to mice alleviated lipopolysaccharide (LPS)-induced inflammatory fever and led to the generation of K2-derived peptides (K2Ps) and their sulfhydrated form (K2P-SSH) in the intestine. Compared with K2Ps, K2P-SSH showed enhanced anti-inflammatory and antioxidant activities and promoted widespread protein sulfhydration. Mechanistically, K2P-SSH induced sulfhydration of high-mobility group box 1 (HMGB1) at Cys23 and Cys106, which was associated with reduced disulfide bond formation and dimerization, impaired interaction with toll-like receptor 4 (TLR4), and attenuation of downstream inflammatory signaling. Taken together, this study reveals a previously unrecognized role of K2 in converting H_2_S into protective sulfhydration signals and identifies K2P-SSH as a bioactive intermediate that alleviates oxidative-inflammatory stress via sulfhydration of HMGB1 at Cys23 and Cys106.

## Graphical abstract

**Figure:**
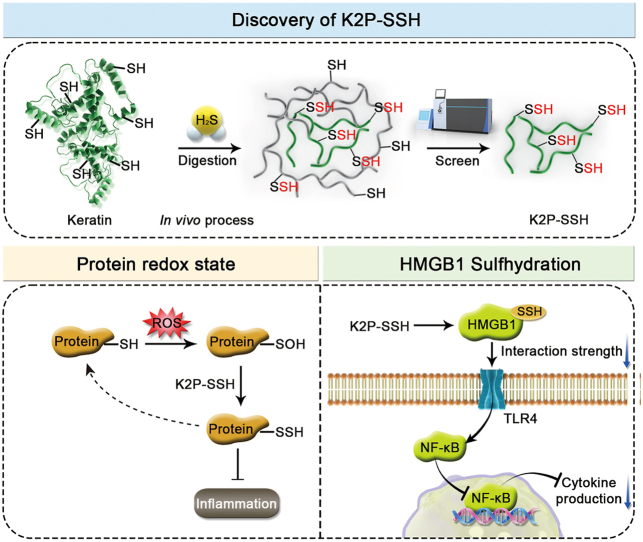

## 1 Introduction

Previous studies have confirmed that the abundant disulfide bonds (-S-S-) and thiol groups (-SH) in keratin play a regulatory role in modulating oxidative-inflammatory stress pathways [1]. These moieties exert diverse biological effects, with a central mechanism involving their participation in sulfur transport, sulfhydration, and the regulation of reactive sulfur species (RSS) [2]. RSS are a class of endogenous bioactive sulfur compounds, including H_2_S, cysteine persulfide (CysSSH), and glutathione persulfide, which are primarily synthesized by enzymes such as cystathionine γ-lyase (CSE), cystathionine β-synthase (CBS), and 3-mercaptopyruvate sulfurtransferase (3-MST) [3]. Under pathological conditions, the levels of these compounds show significant alterations [4,5]. Numerous studies have revealed that RSS participate in key physiological processes, including cellular signaling, redox homeostasis, and metabolic regulation, thereby contributing to anti-inflammatory and antioxidant responses, anti-apoptotic activity, and energy metabolism [3,6].

Sulfhydration is a reversible redox-dependent post-translational modification (PTM) of proteins that predominantly targets cysteine residues. Under physiological or pathological conditions, RSS induce protein sulfhydration, modulating protein activity and interactions with binding partners to influence cellular function [[7], [8], [9]]. A critical protective role of sulfhydration is its ability to prevent excessive oxidative modifications that lead to protein dysfunction. For example, during inflammation or oxidative stress, excessive reactive oxygen species (ROS) can oxidize protein -SH to sulfenic acid (-SOH). Further oxidation may generate sulfinic (-SO_2_H) or sulfonic (-SO_3_H) acid derivatives, both of which are associated with impaired protein function, while sulfonic acid formation is generally considered irreversible [10,11]. In contrast, sulfhydration can convert -SOH to persulfide (-SSH), blocking this cascade of harmful oxidation. Moreover, the -SSH can be reduced back to the active -SH form through cellular reductant systems such as thioredoxin and glutathione, thus restoring protein function. Due to its cytoprotective and regulatory roles, sulfhydration has emerged as a promising therapeutic target in cardiovascular diseases [8], immune disorders [6,12], and cancer [13].

Recent studies on protein sulfhydration under oxidative-inflammatory stress have increasingly focused on key signaling pathways, particularly the Kelch-like ECH-associated protein 1 (Keap1)–nuclear factor erythroid 2-related factor 2 (Nrf2) and nuclear factor kappa-B (NF-κB) axes [14]. It has been demonstrated that sulfhydration of Cys151 and Cys613 in Keap1 promotes its dissociation from Nrf2, thus facilitating increased translocation of Nrf2 into the nucleus. Once there, Nrf2 binds to antioxidant response elements, activating downstream antioxidant pathways and exerting cytoprotective effects [[15], [16], [17]]. In the NF-κB pathway, sulfhydration at Cys38 of p65 enhances its interaction with ribosomal protein S3, leading to upregulation of anti-apoptotic genes [18]. Furthermore, sulfhydration of p65 suppresses NF-κB pathway activation, reduces ROS accumulation, and inhibits inflammasome activation, collectively contributing to the attenuation of inflammatory responses [19]. Under oxidative-inflammatory stress conditions, HMGB1 is actively exported from the nucleus to the cytoplasm and subsequently secreted into the extracellular space, where it acts as a damage-associated molecular pattern molecule to exacerbate disease progression. Studies have confirmed that cysteine residues Cys23, Cys45, and Cys106 in HMGB1 are critically involved in mediating oxidative-inflammatory stress. When these residues form intramolecular or intermolecular disulfide bonds, HMGB1 undergoes conformational changes that significantly increase its binding affinity for TLR4, thereby promoting the release of pro-inflammatory cytokines and amplifying both inflammatory and oxidative responses [20].

Current research on H_2_S-related materials has primarily focused on developing H_2_S donors [21,22], including diallyl trisulfide, GYY4137, and N-benzoylthiobenzamides, which exhibit anti-inflammatory, ROS-scavenging, and cardioprotective effects. However, their clinical application is hindered by poorly controlled H_2_S release kinetics, as accumulation beyond a threshold concentration induces toxicity [23]. Meanwhile, endogenous H_2_S is primarily regulated by enzymes such as CSE, CBS, and 3-MST, yet pharmacological strategies to modulate endogenous H_2_S remain largely unexplored [5].

In this study, we found that KF regulates both H_2_S homeostasis and protein redox state through sulfhydration. Proteomics analysis of keratins identified K2 as the most hyperreactive thiol-containing protein, and further investigation demonstrated its antipyretic activity *in vivo*. Following oral administration to mice, K2 was processed in the intestine to generate K2-derived sulfhydrated peptides (K2P-SSH). In vitro, K2P-SSH promoted widespread intracellular protein sulfhydration in RAW264.7 and bEnd.3 cells. This increase in protein sulfhydration was accompanied by reduced excessive oxidative modifications of cysteine residues, including -SO_2_H and -SO_3_H formation, and enhanced cellular resistance to oxidative stress. Moreover, K2P-SSH induced sulfhydration of HMGB1 at Cys23 and Cys106, which was associated with reduced disulfide bond formation and dimerization, impaired HMGB1–TLR4 interaction, and attenuation of downstream inflammatory signaling. Additionally, K2P-SSH induced sulfhydration of p65 at Cys120 and Keap1 at Cys613. These modifications coincided with reduced p65 activation and nuclear translocation, as well as enhanced Nrf2 nuclear accumulation following dissociation of the Keap1–Nrf2 complex. Collectively, these findings identify K2P-SSH as a bioactive intermediate that promotes anti-inflammatory and antioxidant responses through protein sulfhydration.

## 2 Material and methods

### 2.1 Extraction and preparation of keratin

Rhinoceros horn (Rhinoceri Asiatici Cornu) was purchased from Jiangsu Medicinal Materials Company (Permission State Forestry Administration of China for purchasing Rhino horn samples, 2003, No. 7). *Saiga antelope* horn (Saigae Tataricae Cornu), water buffalo horn (Bubali Cornu) and goat horn (Caprae Hircus Cornu) were purchased from Jiangsu Medicine Company (Jiangsu, China). Yak horn (Bovis Grunniens Cornu) was purchased from a slaughterhouse located on Linkuo North Road in Lhasa, Tibet. All horn products were initially obtained in pieces form, and then were powdered.

Approximately 10 g of horn powder was mixed with 80 mL of 4% sodium dodecyl sulfate (SDS, #3250gR500, Biofroxx, Guangzhou, China) lysis buffer containing 100 mM dithiothreitol (DTT, #YD0021, YIFEIXUE Biotech, Nanjing, China) with or without 0.2 M iodoacetamide (IAA, #ST1411, Beyotime, Shanghai, China), then incubated at 65°C for 5 h. After centrifugation, 60 mL of supernatant was collected. Subsequently, 300 mL of 95% ethanol (#32061, Yasheng Chemical, Wuxi, China) was added, and proteins were precipitated at 4°C overnight. After centrifugation, the supernatant was discarded. The pellet was washed twice with 200 mL of 80% ethanol and then air-dried. These proteins extracted from water buffalo horn were defined as KF and KF-IAA.

### 2.2 Recombinant K2 expression and purification

Recombinant keratin expression and purification were performed following a previously reported method [24]. Briefly, the sequence of P25691 (Keratin, type II microfibrillar) was retrieved from the Uniprot (https://www.uniprot.org/uniprotkb/P25691/entry). Cloning vector pET-22 b (+) was used as a plasmid, and *E. coli* BL21 (#C504-02, Vazyme Biotech Co., Ltd., China) was used as a host strain for expression. The transformed bacteria were first inoculated in Luria Broth (LB, #ST156, Beyotime, China) medium with 100 μg/mL ampicillin (#A100339-0005, Sangon Biotech, Shanghai, China) and incubated overnight at 37°C, 220 rpm. Isopropyl β-d-1-thiogalactopyranoside (IPTG, #A600168-0005, Sangon Biotech, China) was added to a final concentration of 0.5 mM to the LB media with OD 600 of 0.6–0.8. Protein expression was induced at 37°C, 220 rpm for 12 h. Cells were harvested through 8000 rpm centrifugation at 4°C for 15 min and the pellets were stored at −80°C for subsequent purification. Cell pellets were resuspended in different buffers and purified with Ni-column (#SA036025, Smart-Lifesciences, Changzhou, China). The supernatant was collected for SDS-PAGE analysis, dialyzed against water for 3 days, and then lyophilized for storage.

### 2.3 Preparation and characterization of sulfhydrated KF (KF-SSH), sulfhydrated K2 (K2-SSH), K2Ps and K2P-SSH

To simulate the *in vivo* digestion process of K2, the samples were lyophilized using a freeze vacuum dryer (Free Zone, Labconco, USA). 2 g samples were dissolved in 20 mL of artificial intestinal fluid (#R30384, YUANYE, Shanghai, China) and incubated for 4 h at 37°C. After centrifugation, the K2 hydrolysates were collected. Samples were desalted using a C18 column (#WAT043395, Waters Corp., Milford, MA, USA) and concentrated using a vacuum centrifuge (CentriVap, Labconco, USA) to obtain K2Ps.

Protein concentration of K2Ps was determined using a Pierce Quantitative Fluorometric Peptide Assay (#23275, Thermo Scientific, Waltham, MA, USA). The concentration was adjusted to 4 mg/mL using 50 mM Na_2_HPO_4_ (#R27289, YUANYE, Shanghai, China). 5 mL solution was mixed with 3.4 mL of 10 mM 5,5′-dithiobis-(2-nitrobenzoic acid) (DTNB, #YD0032, YIFEIXUE, Nanjing, China) and incubated at 37°C in the dark with rotation for 30 min. Subsequently, 730 μL of 500 mM Na_2_S·9H_2_O (#GB61184, Glpbio, Montclair, CA, USA) was added, and the mixture was incubated at 37°C for another 30 min. The reaction mixture was desalted using a C18 column, dried using a vacuum centrifuge to obtain K2P-SSH. The workflow was illustrated in Fig. S3A. The samples were analyzed by nanoLC-MS/MS method as described in our previous work [1]. KF and K2 were subjected to the same procedure to generate KF-SSH and K2-SSH, with excess Na_2_S removed by ultrafiltration devices (#UFC9003, Millipore, USA).

### 2.4 Identification of various animal-derived keratins

Animal-derived keratin was extracted using the same method as for KF. Protein samples (2 mg/mL) were boiled with 1× loading buffer, subjected to SDS-PAGE. Subsequently, the in-gel digestion was performed as described previously [25]. Briefly, gel pieces were destained using silver destaining solution and ultrapure water until clear. 10 mM DTT was added, and samples were incubated at 56°C for 1 h. Then, 20 mM IAA was added, and samples were incubated at room temperature for 30 min. After washing with 100 mM NH_4_HCO_3_ (#A431332, aladdin, Shanghai, China) and dehydrating with acetonitrile, the gel pieces were dried using a vacuum concentrator. Then, 2 μg of trypsin was added, and digestion was carried out at 37°C overnight. Peptides were extracted using extraction buffer by sonication for 10 min. After centrifugation, the supernatant was collected, desalted using in-house tip columns, and analyzed by nanoLC-MS/MS.

### 2.5 RSS analysis

LC-MS/MS analysis of RSS was performed according to procedures reported previously [5,26,27]. Samples of plasma or tissue homogenates were mixed with a methanol solution containing 5 mM Br-bimane (MBBr; #B860547, Macklin, Shanghai, China) and incubated at 37°C for 15 min 800 μL of acetonitrile (#1.00030.4008, Merck Co., Ltd. Darmstadt, Germany) was added, vortexed thoroughly, and incubated at 4°C for 30 min. After centrifugation, 800 μL of supernatant was collected, dried using a vacuum centrifuge, and reconstituted in 400 μL of 10% acetonitrile. Samples were analyzed using a Triple Quad 6500 + mass spectrometer (AB SCIEX, USA) coupled to a Waters ACQUITY BEH T3 column (2.1 × 100 mm, 1.8 μm). The mobile phase was composed of A (0.1% formic acid in water) and B (0.1% formic acid in acetonitrile). Samples were injected and separated using a gradient elution of 0−1.5 min with 10% A, 1.5−6 min with 90%−5% A, 9−10 min with 5%−90% A, and 10−11 min with 90% A. Column temperature was 40°C, flow rate 0.3 mL/min, and injection volume 2 μL. The eluate was directed to the mass spectrometer for analysis. Table S7 summarizes the multiple reaction monitoring (MRM) parameters for RSS.

### 2.6 Co-incubation of KF with gut microbiota supernatant

Approximately 200 mg of fresh mouse feces was placed in a centrifuge tube with 1 mL of phosphate-buffered saline (PBS, #G4202, Servicebio, Wuhan, China). The mixture was thoroughly homogenized three times at 60 Hz for 60 s each. After centrifugation at 200*g* for 20 min, the supernatant was diluted 100-fold into chopped meat broth liquid medium (#LA4610, Solarbio, Beijing, China) and incubated in an anaerobic pouch (#AN0025A, Thermo Scientific, USA) for 2 days. Following centrifugation at 14000*g* for 10 min, the supernatant (gut microbiota supernatant) was collected. Approximately 4 mg of KF was dissolved in 1 mL of ultrapure water by vortexing. After centrifugation, 500 μL of the KF supernatant was mixed with 500 μL of the microbiota supernatant and incubated with rotation for 30 min.

### 2.7 H_2_S consumption assay

The determination of free thiol was performed according to previously reported methods [28]. The free thiol concentrations of cysteine and KF were quantified.

Aliquots (100 μL) of cysteine or KF samples containing varying free thiol concentrations were co-incubated with 400 μL of gut microbiota suspension for various time points. The samples were then centrifuged at 12,000 rpm, and 400 μL of the supernatant was collected and mixed with 1.2 mL of acetonitrile containing 5 mM MBBr. After thorough mixing and centrifugation, 1.5 mL of the supernatant was evaporated to dryness and reconstituted in 1 mL of 10% acetonitrile. The reconstituted samples were diluted 1000-fold and analyzed by LC-MS/MS. In parallel, 100 μL of KF solution adjusted to the indicated free thiol concentration was incubated with 400 μL of 100 μM Na_2_S for the indicated time periods. Samples were processed as described above, and H_2_S levels were quantified by LC-MS/MS as described in the RSS analysis section.

### 2.8 Reducible covalent capture of purification (RCCP) method for determining putatively sulfhydrated proteins

The RCCP method was performed as previously reported with slight modifications [29]. For cell-based experiments, intact cells were first stimulated with H_2_O_2_, LPS, or TNF-α for 4 h, followed by incubation with K2P-SSH or KF-SSH where applicable. After treatment, cells were washed with ice-cold PBS and lysed to obtain total cellular protein samples. For tissue- or protein-based experiments, tissue homogenates or protein samples were directly prepared. Protein concentration was determined using a BCA kit (#GK10009, Glpbio, Montclair, CA, USA) and adjusted to 3 mg/mL. Briefly, 100 μL of SulfoLink coupling resin (#20401, Thermo Scientific, USA) was washed twice with PBS and incubated with 1 mL of protein sample at 25°C for 1 h with gentle agitation. After removal of the supernatant, the resin was washed twice with 1 mL of 2% SDS and subsequently boiled in fresh 2% SDS for 5 min. After centrifugation, captured proteins were eluted with 40 μL of 2× loading buffer (#BL529A, Biosharp, Hefei, China) containing DTT at 100°C for 5 min. The supernatant was collected after centrifugation for subsequent SDS-PAGE analysis.

For LC-MS/MS identification, bead-bound proteins were digested with trypsin (#V5113, Promega, Fitchburg, WI, USA) overnight at 37°C. The beads were washed three times with ultrapure water, and peptides were eluted with 25 μL of DTT (200 mM) for 1 h at 25°C. After adding 25 μL of 100% methanol, the supernatant was desalted using in-house tip columns, dried using a vacuum centrifuge, and reconstituted in 20 μL of 0.1% formic acid (FA; #85170, Thermo Scientific, USA). Following centrifugation, 10 μL of the supernatant was transferred to a vial for nanoLC-MS/MS analysis.

Although the RCCP method has been widely used for enrichment of sulfhydrated proteins, it should be noted that proteins that are not sulfhydrated, but are bound through disulfide bonds to those retained in the resin (either sulfhydrated or containing free thiol groups), can be eluted by DTT treatment. Therefore, proteins identified by this method are referred to as putatively sulfhydrated proteins unless further validated by independent approaches.

### 2.9 Sulfhydration assay using Fluorescence-PAGE

Cells were first stimulated with H_2_O_2_, TNF-α, or LPS for 4 h, followed by treatment with K2P-SSH or KF-SSH where applicable. Cells were then washed with PBS and lysed to obtain protein samples. Cell lysates were collected and incubated with 1 mM IA-Rhodamine probe (#1281660, J&K Scientific, Beijing, China) at room temperature in the dark with rotation for 1 h. For total protein sulfhydration assay, cell lysates were treated with or without DTT (1 mM) at 4°C in the dark for 1 h. The samples were boiled and subjected to electrophoresis, and then scanned with imaging system (excitation 520 nm, 206-150-001, baygene, Beijing, China). The fluorescence intensity was quantified using ImageJ software.

### 2.10 Detection of sulfenic acid (-SOH)

The levels of -SOH in proteins were determined as previously described [30,31]. Cells were exposed to 1 mM H_2_O_2_ and then incubated with 100 μM DCP-Bio1 (#EE0028, Kerafast, USA), a biotinylated cysteine sulfenic acid (R-SOH) probe. The lysis buffer consisted of NP-40 (#P0013F, Beyotime, China) containing 100 μM ethylene diamine tetraacetic acid (EDTA, #BL518A, Biosharp, China), 10 mM N-ethylmaleimide (#HY-D0843, MCE, Shanghai, China), 10 mM IAA, and a protease inhibitor cocktail. After washing with PBS and homogenizing in lysis buffer, cell lysates were applied to ultrafiltration devices (#UFC5010, Millipore, USA) to remove excess DCP-Bio1. Protein concentration was determined by BCA assay. 1.5 mg of protein was incubated with streptavidin beads overnight at 4°C. After centrifugation, the beads were washed twice, sequentially with 1% SDS, 4 M urea, 1 M NaCl, and ultrapure water. Affinity captured proteins were eluted with loading buffer and subjected to SDS-PAGE.

### 2.11 Quantitative thiol reactivity profiling (QTRP) assays

QTRP studies were performed following previously reported methods [32,33]. Protein concentration was determined and adjusted to 3 mg/mL after cells were collected and lysed. 500 μL of cell lysate was tagged with 100 μM alkynyl IAA (IPM, #EVU111, Kerafast, Boston, MA, USA) at room temperature for 1 h. Then, 800 mM IAA was added, and the mixture was incubated in the dark for 1 h. Proteins were precipitated using the methanol-chloroform method. 20 μg of trypsin was added for overnight digestion. IPM probe-modified peptides were tagged with isotopically labeled azido-biotin reagents with a photo-cleavable linker (#EVU102, #EVU151, Kerafast) via copper-catalyzed azide-alkyne cycloaddition (CuAAC). The reaction solution was added to 200 μL of streptavidin agarose beads (#20347, ThermoFisher Scientific, USA) and incubated in the dark with rotation for 2 h. Beads were washed twice sequentially with 50 mM NaOAc (#C0130694023, Nanjing Reagent, Nanjing, China), pH 4.5, and 50 mM NaOAc, pH 4.5, containing 2 M NaCl, then ultrapure water with rotation to remove nonspecifically bound peptides. Beads were resuspended in 1.2 mL NH_4_HCO_3_ (25 mM) and subjected to 365 nm UV light for 2 h at room temperature. After centrifugation, the supernatant was collected, dried using a vacuum concentrator, desalted using in-house zip-tip columns. Photo-released peptides were identified and quantified by nanoLC-MS/MS.

Raw MS data files were analyzed using PEAKS Studio 8.5 software. Database search was performed against the Bovine reviewed database or the Mouse reviewed database (UniProt). Precursor-ion mass tolerance was set to 20.0 ppm, fragment mass tolerance to 0.1 Da. Enzyme specificity: Trypsin, maximum missed cleavages: 2. Variable post-translational modifications included: IPM-tagged sulfhydrates (+284.09, light, +290.09, heavy), IPM-tagged thiols (+252.12, light, +256.12, heavy), thiols (+252.12). A maximum of 4 modifications were allowed per peptide.

### 2.12 Dimethyl-labeling-based strategy quantitative proteomics

To determine and rank the reactivity of all -SH groups in peptides derived from KF and KF-SSH digested in artificial intestinal fluid, we employed a dimethyl-label-based quantitative strategy adapted from the QTRP method with slight modifications (Fig. 2A). For peptide reactivity assessment, the isotopically labeled azido-biotin reagents were replaced with UV-cleavable azido-biotin (#BCC-30, Confluore, Xian, China). After washing beads twice with ultrapure water, beads were washed with 100 mM triethyl-ammonium bicarbonate buffer (TEAB, #T466164, aladdin, China). Beads were then dimethyl-labeled as previously described [34]. Peptides labeled with 10 μM IPM were tagged with HCHO as the light-labeled group, and peptides labeled with 100 μM IPM were tagged with CD_2_O as the heavy-labeled group. Subsequent steps were identical to QTRP assays. Data were analyzed using the same QTRP parameters, with variable post-translational modifications including dimethyl (+28.03, light), dimethyl (+32.06, heavy), and iodoacetamide alkylation (+57.02).

### 2.13 Lead acetate assay to detect H_2_S production

H_2_S production capacity was measured by the lead acetate method [5]. Briefly, K2 and K2-SSH concentrations were measured using a BCA kit and then diluted with PBS to the indicated concentrations. Na_2_S was similarly diluted with PBS to the indicated concentrations. 180 μL of the solution was placed into each well of a 96-well plate. The plate was then overlaid with lead acetate-embedded filter paper and incubated at 37°C until lead sulfide formation. Quantification was performed by densitometry analysis using ImageJ (version: 1.51n) with the IntDen function.

### 2.14 Mice and housing conditions

C57BL/6 J mice (8–10 weeks old) were purchased from Jiangsu Huachuangxinnuo Medical Technology Co., Ltd. (Taizhou, China). Animal experiments were approved by the Institutional Animal Care and Use Committee of Nanjing University of Chinese Medicine (#202408A041, #202410A029, #202501A062, Nanjing, China). Mice were provided with *ad libitum* chow and drinking water and maintained on a 12 h light/dark cycle at 24 ± 2°C and 40%–60% relative humidity. All animal experiments were performed in strict accordance with the regulations of the Animal Ethics Committee of Nanjing University of Chinese Medicine.

### 2.15 Antipyretic activity test

The remote thermometry was conducted as previously described [35]. A temperature-sensing capsule (#P022-A, Anipill capsule, France) was implanted into the abdominal cavity of each mouse. Core body temperature was monitored in real-time using a capsule monitor (#040-L, Anilogger monitor, France). One week post-surgery for recovery, mice received an intravenous tail vein injection of 50 μg/kg lipopolysaccharide (LPS, #L2880, Sigma-Aldrich, St. Louis, MO, USA) and were simultaneously administered aspirin (200 mg/kg, #BJ71772, Bayer, Germany), K2 (0.09 g/kg, 0.18 g/kg), K2-IAA (0.18 g/kg) and KF (1.8 g/kg) via oral gavage. Real-time body temperature (Tr) was monitored continuously for 8 h post-LPS injection and treatment using the Anipill system. The mean real-time temperature measured 0.5 h before LPS injection was calculated as the basal body temperature (Tb). The difference (ΔT = Tr−Tb) between real-time temperature (Tr) and basal temperature (Tb) was calculated and plotted over time. The area under the curve (AUC) for ΔT between 1 h and 5 h post-LPS injection was calculated and presented in bar graphs.

### 2.16 RNA-seq and data analysis

RNA was extracted and purified from mouse small intestinal tissue for next-generation paired-end sequencing on an Illumina sequencing platform. Library construction and sequencing were performed by Suzhou PANOMIX Biomedical Technology Co., Ltd. (Suzhou, China). Differential gene expression analysis (p-value < 0.05) was performed using the company's cloud platform (https://www.biodeep.cn/). Gene set enrichment analysis (GSEA) was performed using the Java GSEA Desktop Application with default parameters and the C5 ontology gene sets, which are provided as part of MSigDB V6.2 (https://www.gsea-msigdb.org/gsea/msigdb/index.jsp) as default screening gene sets.

### 2.17 Measurement of Interleukin-1β (IL-1β), Interleukin-6 (IL-6), tumor necrosis factor-α (TNF-α), HMGB1

Concentrations of IL-1β (#JX-2776A1), IL-6 (#JX-2899A1), TNF-α (#JX-2868A1), and HMGB1 (#JX-5840A1) in mouse plasma were determined using Sandwich enzyme-linked immunosorbent assay (ELISA) kits (Yancheng Junxing Biotechnology Co., Ltd.) according to the manufacturer's instructions.

### 2.18 Artificial gastric and intestinal fluid simulated the digestion of KF

KF (150 mg) was mixed with 1 mL of artificial gastric fluid (#N21IR19841A, YUANYE, Shanghai, China) and incubated at 37°C for 15 min. After centrifugation, the pellet was resuspended in 500 μL of artificial intestinal fluid and incubated at 37°C for 15, 30, 60, 120, or 240 min, respectively. The reaction was terminated by the addition of 40 μL of 10% trifluoroacetic acid (TFA, #T818781, Macklin, Shanghai, China), followed by centrifugation. After discarding the supernatant, 600 μL of 4% SDS was added, and the samples were vortexed thoroughly to ensure complete dissolution before SDS-PAGE analysis.

### 2.19 Peptide P1 analysis

Peptide P1 (TFSSCSAVAPK) derived from P25691 was chemically synthesized by GenScript Corporation (Nanjing, China). Mice were administered 2 mg peptide by oral gavage, intraduodenal injection, or intraperitoneal injection. Blood samples were collected 30 min after dosing and centrifuged at 12,000 rpm for 10 min. An aliquot of 100 μL of serum was mixed with 400 μL of methanol, vortexed thoroughly, and centrifuged. 450 μL of the supernatant was collected and evaporated to dryness. The sample was reconstituted in 100 μL of 10% acetonitrile. After centrifugation, 10 μL of supernatant was transferred to vials for LC-MS/MS analysis. Table S7 summarizes the MRM parameters for P1.

### 2.20 Cell culture

RAW264.7 macrophages and bEnd.3 mouse brain endothelial cells were purchased from Procell Life Science & Technology Co., Ltd. (Wuhan, China). HEK293T cells were obtained from the American Type Culture Collection (ATCC, USA). All cell lines were cultured in DMEM medium (#PM150210, Procell, China) supplemented with 10% fetal bovine serum (FBS; #164210-50, Procell, Wuhan, China) and 1% penicillin-streptomycin (#C100C5, NCM Biotech, Suzhou, China), maintained at 37°C in a humidified incubator in the presence of 5% CO_2_.

Cells were passaged at approximately 80% confluency. bEnd.3 cells were seeded at 2 × 10^5^/mL. After 24 h, cells were treated with K2P-SSH (6.25, 12.5, or 25 μg/mL) or K2Ps (25 μg/mL) for 12 h based on preliminary cytotoxicity test results. Control cells received fresh medium. Subsequently, cells were treated with 1.1 mM H_2_O_2_ (#C0404510123, Nanjing Reagent, China) and 100 ng/mL TNF-α (#GP20968, Glpbio, USA) for 4 h. RAW264.7 cells were seeded at 2–3×10^5^/mL. After 24 h, cells were treated with K2P-SSH (12.5, 25 or 50 μg/mL) or K2Ps (50 μg/mL) for 12 h. Then, the cells were treated with 50 ng/mL LPS for 4 h.

The treatment sequence was selected according to the objective of each experiment. For experiments investigating the biological effects of K2P-SSH, cells were pretreated with K2P-SSH before stimulation. In contrast, for experiments designed to assess protein sulfhydration, KF-SSH or K2P-SSH was administered after stimulation to facilitate detection of sulfhydration.

### 2.21 Free radical scavenging activity of K2P-SSH

1,1-diphenyl-2-picrylhydrazyl‌ (DPPH) radical scavenging activity was measured as described previously [36]. Briefly, a DPPH solution was prepared by dissolving DPPH (#D807297, Macklin, China) in ethanol at a concentration of 0.2 mM. An aliquot (500 μL) of sample solution at each concentration was mixed with 500 μL of DPPH solution and incubated at room temperature for 30 min. The UV-Vis absorbance of the mixtures was measured at 517 nm using a microplate reader (Multiskan GO, Thermo, USA). Hydroxyl radical scavenging assay was measured as described previously [37]. The sample solution was mixed sequentially with 10 mM FeSO_4_ (#F903428, Macklin, China) and 100 mM H_2_O_2_, and incubated at room temperature for 10 min. Then, 10 mM salicylic acid in ethanol (#S817529, Macklin, China) was added, and the mixture was incubated at room temperature for 30 min. The absorbance was determined at 510 nm using a microplate reader.

The reducing power assay was performed as previously reported [38]. Briefly, 0.5 mL of phosphate buffer (0.2 M, #R26289, YUANYE, China) and 0.5 mL potassium ferricyanide (1.0%, w/v, #A800764, Macklin, China) were added to 0.2 mL of sample. The mixture was vortexed and incubated at 50°C for 20 min. Then, 0.5 mL of trichloroacetic acid (10%, w/v, #T818878, Macklin, China) was added. After centrifugation, 0.5 mL of ultrapure water and 0.2 mL of ferric chloride (0.1%, w/v, #I811935, Macklin, China) were added to 0.5 mL of supernatant. The mixture was incubated at room temperature for 10 min and the absorbance was determined at 700 nm using a microplate reader.

The free radical scavenging activity of samples was calculated using the following equation:

$$\text{Free}\;\text{radical}\;\text{scavenging}\;\text{activity}\;\left(\%\right)=\left(\frac{\Delta A\;\text{of}\;\text{control}-\Delta A\;\text{of}\;\text{sample}}{\Delta A\;\text{of}\;\text{control}}\right)\times 100\%$$

(ΔA of control represents the absorbance of the control groups. ΔA of sample represents the absorbance of the sample groups).

### 2.22 Flow cytometry

Cells were washed three times with PBS and collected. After centrifugation, cells were resuspended in 1 mL DMEM medium containing 10 μM 2′,7′-dichlorofluorescein diacetate (DCFH-DA) probe (#S31479, YUANYE, China) and incubated in the dark at room temperature with rotation for 30 min. Cells were washed with PBS, centrifuged at 800 g for 5 min, resuspended in 200 μL PBS, and strained through a 40-μm cell strainer. Intracellular ROS levels were analyzed using a flow cytometer (MoFlo XDP, BECKMAN COULTER, USA). Data were analyzed using FlowJo Version 10 software.

### 2.23 Western blot

Antibodies against HMGB1 (#10829-1-AP), p65 (#10745-1-AP), phospho-p65 (#82335-1-RR), Nrf2 (#16396-1-AP), superoxide dismutase 1 (SOD1, #10269-1-AP), Keap1 (#10503-2-AP), TLR4 (#19811-1-AP), heme oxygenase 1 (HO-1, #10701-1-AP), and HRP-conjugated goat anti-rabbit IgG (H + L) (#SA00001-2) were obtained from Proteintech (Wuhan, China). The cyclooxygenase-2 (COX-2) antibody was obtained from Zenbio (#501253), and the Flag-Tag (#30503ES60) was obtained from Yeasen Biotechnology (Shanghai, China).

Cells were washed twice with cold PBS and lysed with RIPA supplemented with a protease inhibitor cocktail (#P1005, Beyotime, China). The soluble proteins were collected, and their concentrations were measured using a BCA kit. Proteins were resolved by SDS-PAGE and transferred to PVDF membranes (#IPVH00010, Merck, Germany). Membranes were blocked with rapid protein blocking buffer (#G2052, Servicebio, China), incubated with primary antibodies overnight at 4°C, followed by the corresponding horseradish peroxidase-labeled secondary antibodies for 2 h. ECL Kit (#FD8000, Fude Biotech, Hangzhou, China) was applied according to the manufacturer's protocols. Images were captured by the Tanon 4200 Chemiluminescent Imaging System (Tanon, Shanghai, China), and the signal intensity was quantified using ImageJ software.

### 2.24 Quantitative real-time polymerase chain reaction (qRT-PCR) analysis

Total RNA was extracted from cells using RNA isolator (#RC202, Vazyme Biotech Co., Ltd., China), followed by reverse transcription with HiScript III RT SuperMix (#R223, Vazyme Biotech Co., Ltd., China). Gene-specific primers and ChamQ SYBR qPCR Master Mix (#Q321-02, Vazyme Biotech Co., Ltd., China) were used to quantify mRNA levels of indicated genes. Real-time PCR reactions were performed in triplicate on a CFX Connect™ Real-Time System (Bio-Rad Laboratories, Hercules, CA, USA). The primer sequences used are listed in Table S1.

### 2.25 Immunofluorescence (IF)

Cells were fixed on coverslips for 15 min with 4% paraformaldehyde (#G1101, Servicebio, China) in PBS, washed three times, and permeabilized for 20 min with 0.5% Triton X-100 (#ST1722, Beyotime, China). After three rinses, cells were blocked with 5% bovine serum albumin (BSA, #YB0006, YIFEIXUE Biotech, China) at room temperature for 30 min and treated with the primary antibody (HMGB1, p65, Nrf2, TLR4) at 4°C overnight. Secondary antibodies (#ab150079, Abcam, Cambridge, UK) were incubated with cells for 2 h. After staining with 4′,6-diamidino-2-phenylindole (DAPI, #C1002, Beyotime, China), coverslips were mounted and examined using a confocal microscope (Nikon AX, Japan).

Intracellular H_2_S and -SSH levels were evaluated using the fluorescent probes WSP-5 (#V13160, YUANYE, China) and SSP4 (#SB10, DOJINDO, Japan), respectively. After fixing cells, the cells were loaded with the probes (50 μM WSP-5 or 20 μM SSP4) in the presence of 500 μM hexadecyltrimethylammonium bromide (CTAB, #H811117, Macklin, China) for 30 min, at 37°C, in the dark. After three rinses, cells were stained with DAPI and examined with a confocal microscope.

### 2.26 GSH/GSSG assay

GSH and GSSG levels in cell samples were measured using a commercial assay kit (#A061-1-2, Nanjing Jiancheng Bioengineering Institute, China) according to the manufacturer's instructions. Total glutathione (T-GSH) and oxidized glutathione (GSSG) were quantified at 405 nm using a microplate reader, and reduced glutathione (GSH) levels were calculated as follows: [GSH] = [Total Glutathione] − 2 × [GSSG].

### 2.27 Generation of site mutant construct

HMGB1, p65, Keap1 expression constructs (BOLAZ Biotechnology Co., Ltd., Suzhou, China) were used as templates to generate site mutant construct according to the results of sulfhydration proteomics (HMGB1: C23S, C106S, p65: C105S, C120S, C216S, Keap1: C489S, C613S) using a QuickChange site-directed mutagenesis kit (#114458-99-0, J&K Scientific, China). The mutant constructs were confirmed by DNA sequencing. The primers used to generate point mutant constructs were as follows (Table S2).

RAW264.7 cells were transduced with lentiviral vectors encoding full-length mouse p65^WT^, p65^C120S^, Keap1^WT^, Keap1^C613S^, HMGB1^WT^, HMGB1^C23SC106S^ fused with a puromycin resistance gene (Table S2). The lentiviral vectors were obtained from General Biol Co., Ltd. (Chuzhou, China) and cells were transduced according to the manufacturer's protocols.

### 2.28 Cell transfection

HMGB1, p65, Keap1 overexpressing plasmids were synthesized by BOLAZ Biotechnology Co., Ltd. (Suzhou, China). The used primers sequences were shown in Table S2. Transfections were performed with Lipo 293™ transfection reagent (#C0521, Beyotime, China) according to the manufacturer's recommendations. Targeted protein expression was measured by Western blot 48 h after transfection.

### 2.29 Molecular docking

The interaction between TLR4/myeloid differentiation-2 (MD-2) and HMGB1 was predicted using ZDOCK (version 3.0.2). Protein structures of TLR4/MD-2 and HMGB1 were obtained from the Protein Data Bank. Docking models were ranked according to ZDOCK scores, with higher scores indicating stronger predicted protein–protein interaction. Representative docking conformations were selected based on clustering analysis and docking scores. Protein preparation and visualization were performed using PyMOL 2.6 (The PyMOL Molecular Graphics System).

### 2.30 Co-immunoprecipitation (CO-IP) pulldown assays

Flag pulldown assays were performed as previously described [39]. RAW264.7 cells stably expressing Flag-HMGB1^WT^ or Flag-HMGB1^C23SC106S^ were treated with K2P-SSH and LPS as described above. The cells were washed with ice-cold PBS and lysed in Co-IP lysis buffer (Beyotime) on ice. Then, the cell extracts were immunoprecipitated with anti-FLAG Agarose Gel (#P2202S, Beyotime, China) at 4°C overnight. The immunoprecipitates were thoroughly washed with lysis buffer five times, eluted with SDS loading buffer, boiled at 99°C for 5 min, and then immunoblotted with the indicated antibodies. The samples were incubated with primary antibodies against TLR4, Flag-Tag and β-actin.

### 2.31 Measurement of HMGB1 dimerization

To assess HMGB1 dimerization [20], recombinant HMGB1 (#HY-P700182AF) was obtained from MCE. HMGB1 protein (100 ng/μL) was incubated in normal mouse serum containing 50 ng/mL LPS, 50 μM H_2_O_2_ and 5 mM DTT, followed by treatment with 1 mg/mL K2P-SSH for 45 min at 37°C. Test samples were 20-fold diluted and 100 ng of protein was loaded for Western blot analysis under non-denaturing conditions without β-mercaptoethanol (#BL511A, Biosharp, China).

To detect HMGB1 dimerization in serum, the mice were intraperitoneally injected with 1 mg/kg LPS. After 12 h, K2 (0.18 g/kg) was administered by oral gavage. Serum samples were collected 8 h after the K2 administration. HMGB1 dimerization was detected in 10-fold diluted serum using non-reducing SDS-PAGE.

### 2.32 Surface plasmon resonance (SPR) analysis

To calculate the dissociation equilibrium constant (KD) of HMGB1 and HMGB1^C23SC106S^ binding to TLR4/MD2 (#3146-TM/CF, R&D Systems), SPR analysis was performed as previously described [19] using a Reichert 4SPR instrument (Reichert, USA). Recombinant HMGB1^C23SC106S^ was obtained from General Biol Co., Ltd., Chuzhou, China. Briefly, TLR4/MD2 was immobilized onto a gold sensor slide (#13206066, Reichert, USA) and blocked with 1 M ethanolamine (#E0135, Sigma-Aldrich, USA). HMGB1 proteins were diluted to the indicated concentrations in PBST buffer (1×PBS, 0.1% Tween-20) and injected over the sensor surface to evaluate binding. Real-time sensorgrams were recorded and analyzed using the instrument TraceDrawer software to calculate binding kinetics and KD values.

### 2.33 Statistical analysis

All statistical analyses were performed using GraphPad Prism 9.5 (San Diego, CA, USA). Bioinformatics analyses were conducted using R (http://www.R-project.org/). The number of biological replicates for each experiment is indicated in the corresponding figure legends. Unless otherwise specified, data are presented as mean ± standard deviation (SD) from at least three independent biological replicates. Statistical tests were selected based on sample size and the results of normality testing. For datasets with sample sizes ≤4 per group or those that did not satisfy normality assumptions, non-parametric tests were applied. Comparisons between two groups were performed using either a two-tailed Student's t-test or Mann–Whitney *U* test, whereas comparisons among multiple groups were analyzed using either one-way ANOVA or the Kruskal–Wallis test, as appropriate.

## 3 Results

### 3.1 KF regulates H_2_S homeostasis and protein redox state through sulfhydration

**Fig. 1:**
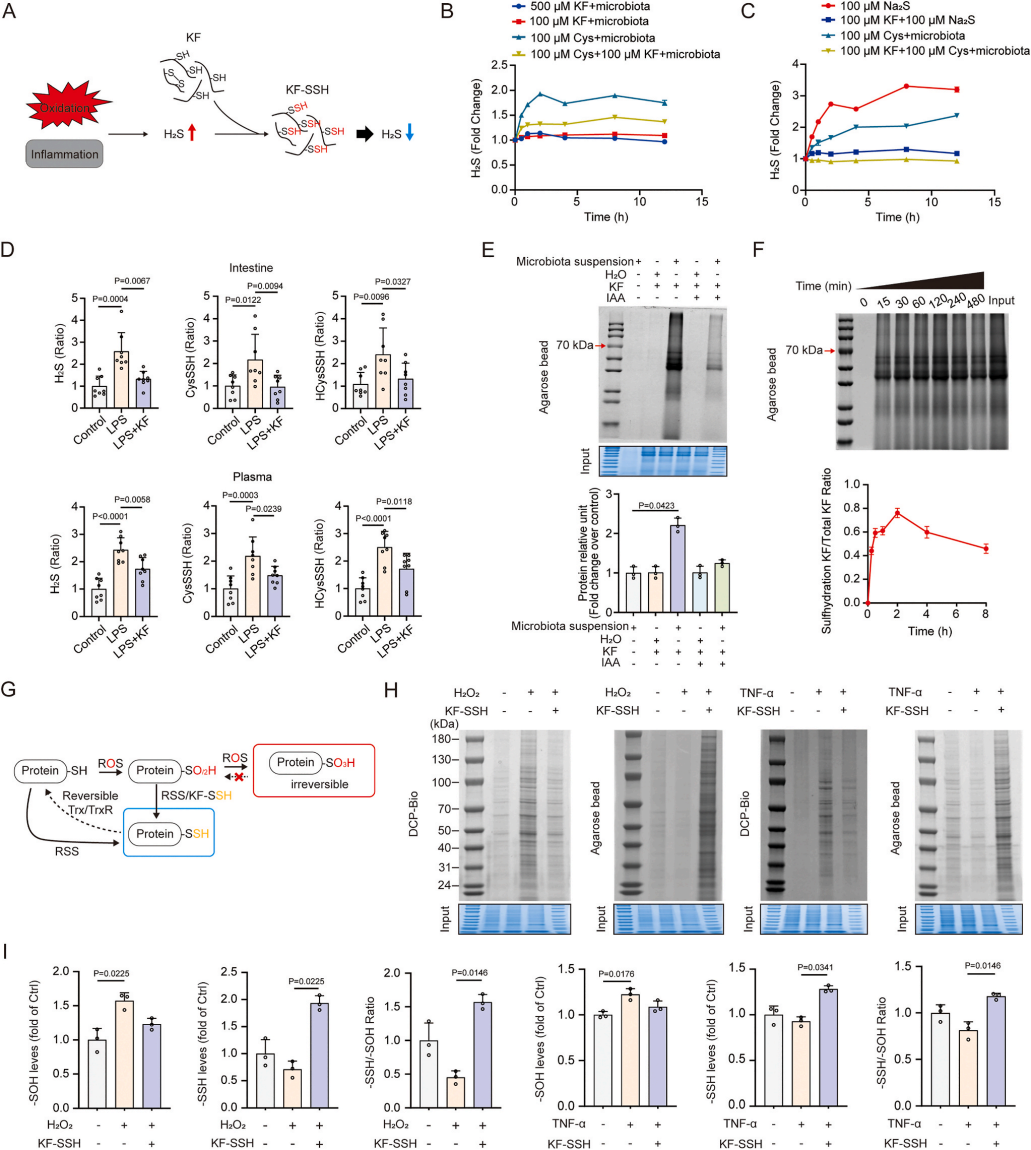
**KF regulates protein redox status through sulfhydration.** (A) Schematic illustration showing that under conditions of oxidative-inflammatory stress, KF reacts with H_2_S to form sulfhydrated KF (KF-SSH), thereby reducing excessive H_2_S accumulation and contributing to H_2_S homeostasis. (B, C) Time-course analysis of H_2_S levels *in vitro*. Gut microbiota cultures were co-incubated with cysteine or KF, while KF was directly incubated with Na_2_S. H_2_S levels were measured by LC-MS. The concentrations of cysteine and KF were normalized based on free thiol (-SH) content. All concentrations shown in the figure represent final concentrations in the reaction mixtures. (D) LC-MS analysis of CysSSH, HCysSSH, and H_2_S levels in the intestine tissues and plasma collected 5 h after oral KF administration (1.8 g/kg, n = 8). (E) RCCP-based analysis of KF sulfhydration following co-incubation of KF (2 mg) with microbiota supernatant for 30 min (n = 3). (F) Time-course analysis of KF sulfhydration following co-incubation with microbiota supernatant for the indicated times using the RCCP method. Input represents the total KF sample prior to RCCP enrichment and was used for normalization of sulfhydration levels (n = 3). (G) Schematic diagram of protein oxidation and sulfhydration. Protein sulfhydration can serve as a protective mechanism against excessive oxidation. (H, I) Detection of intracellular protein sulfenylation and putatively sulfhydrated proteins in bEnd.3 cells. Cells were stimulated with H_2_O_2_ or TNF-α for 4 h, followed by KF-SSH treatment (1 mg/mL) for 1 h before cell lysis. Protein sulfenylation was detected using the DCP-Bio1 probe, whereas putatively sulfhydrated proteins were analyzed by RCCP-based enrichment (n = 3). Data are presented as mean ± SD. Kruskal–Wallis test followed by Dunn's multiple comparisons was performed for **B, E, H,** and for intestinal H_2_S levels in **D**. Mann–Whitney *U* test was performed for **C**. One-way ANOVA followed by Dunnett's multiple-comparison was performed for **D**.

It has been reported that the majority of intestinal H_2_S is produced by the gut microbiota in mice [3,40]. Given that KF is rich in -SH/-S-S- moieties, we hypothesized that these moieties might react with H_2_S. Based on this, we established an *in vitro* culture system of mouse gut microbiota and measured H_2_S levels by LC-MS. Supplementation with 100 μM cysteine (measured as free -SH content) tended to increase H_2_S levels, whereas supplementation with KF at equivalent -SH concentrations (100 μM, or 500 μM) did not appear to increase H_2_S accumulation in gut microbiota cultures. Furthermore, compared with microbiota cultures supplemented with 100 μM cysteine alone, the addition of 100 μM KF tended to result in lower H_2_S levels (Fig. 1B). As shown in Fig. 1C, both 100 μM Na_2_S solution (a rapidly released H_2_S donor) and supplementation of gut microbiota cultures with 100 μM cysteine were associated with increased H_2_S levels, whereas co-incubation with KF tended to reduce H_2_S accumulation under both conditions. Overall, these observations were consistent with the notion that KF does not promote microbiota-derived H_2_S generation and may contribute to H_2_S consumption.

**Fig. 2:**
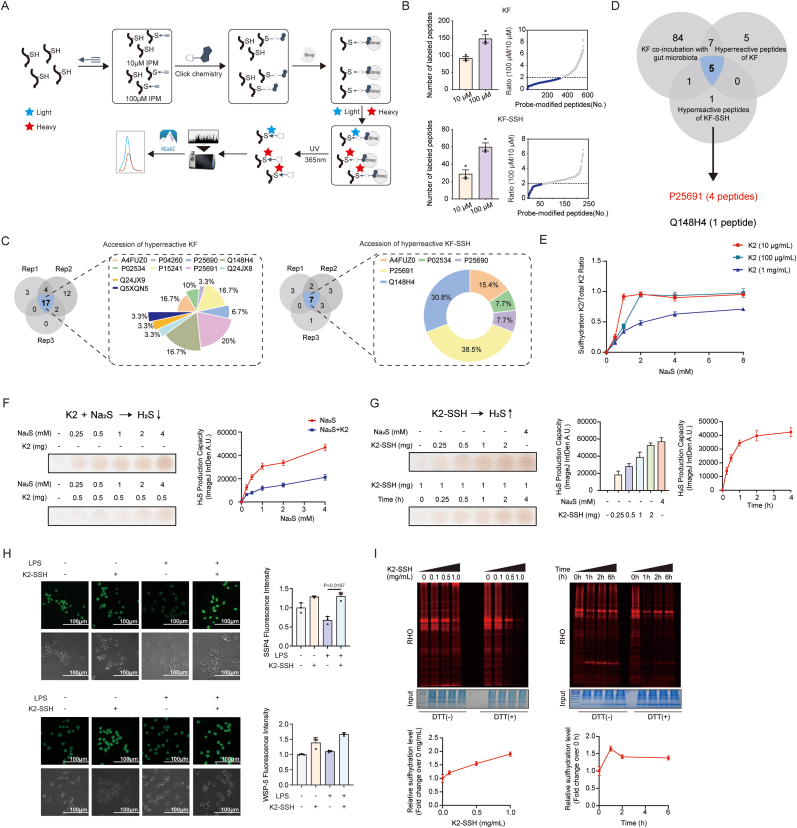
**K2 contains the greatest number of hyperreactive -SH and -SSH, thus maintaining H_2_S homeostasis.** (A) Workflow of a dimethyl-label–based quantitative proteomics strategy for reactivity profiling of -SH and -SSH. (B) Number and ratio of probe-labeled peptides at different probe concentrations in the KF and KF-SSH groups. Peptides labeled with 10 μM IPM were tagged with HCHO as the light-labeled group, and peptides labeled with 100 μM IPM were tagged with CD_2_O as the heavy-labeled group (n = 3). (C) Proteins (Uniprot accession) corresponding to hyperreactive -SH peptides identified in the KF and KF-SSH groups (n = 3). (D) Venn diagram analysis of sulfhydrated peptides derived from KF co-incubation with microbiota supernatant and hyperreactive -SH peptides identified in the KF and KF-SSH groups. (E) Quantification of K2 sulfhydration levels using the RCCP method following treatment with the indicated concentrations of Na_2_S for 2 h (n = 3). (F) H_2_S-consuming capacity of K2 following co-incubation with Na_2_S for 2 h, assessed using the lead acetate method (n = 3). (G) H_2_S production capacity of K2-SSH assessed by the lead acetate method following 2 h incubation (upper panel) or incubation for the indicated times (lower panel, n = 3). (H) Representative fluorescence images of RAW264.7 cells treated with K2-SSH (1 mg/mL) for 1 h and stained with SSP4 (20 μM) or WSP-5 probe (50 μM) to detect intracellular -SSH and H_2_S levels, respectively (n = 3, Scale bar: 100 μm). (I) Detection of sulfhydrated proteins in RAW264.7 cells using the IA-Rhodamine probe. Cells were treated with K2-SSH at indicated times and doses, followed by cell lysis and IA-Rhodamine labeling. $\text{Level}\;\text{of}\;\text{sulfhydration}=\frac{\text{Rhodamine}\;\text{intensity}\;\left(\text{Before}\;\text{DTT}\right)-\text{Rhodamine}\;\text{intensity}\;\left(\text{After}\;\text{DTT}\right)}{\text{Rhodamine}\;\text{intensity}\;\left(\text{Before}\;\text{DTT}\right)}$ (n = 3). Data are presented as mean ± SD. Mann–Whitney *U* test was performed for **B, F**. Kruskal–Wallis test followed by Dunn's multiple comparisons was performed for **H**.

Given that RSS are closely linked to sulfur metabolism and other key physiological activities in the intestinal environment [3,41], we next sought to elucidate the regulatory effects of KF on major RSS in mouse intestines and plasma. To this end, we employed targeted LC-MS to quantify representative RSS, including H_2_S, CysSSH, and homocysteine persulfide (HCysSSH), in mouse intestines and plasma. In LPS-induced febrile mice, the levels of H_2_S, CysSSH, and HCysSSH were elevated, whereas KF oral administration reduced all three sulfur species in both intestinal tissues and plasma (Fig. 1D). These findings suggest that KF modulates sulfur homeostasis, potentially by counteracting excessive H_2_S accumulation under inflammatory conditions.

**Supplementary Fig. 1:**
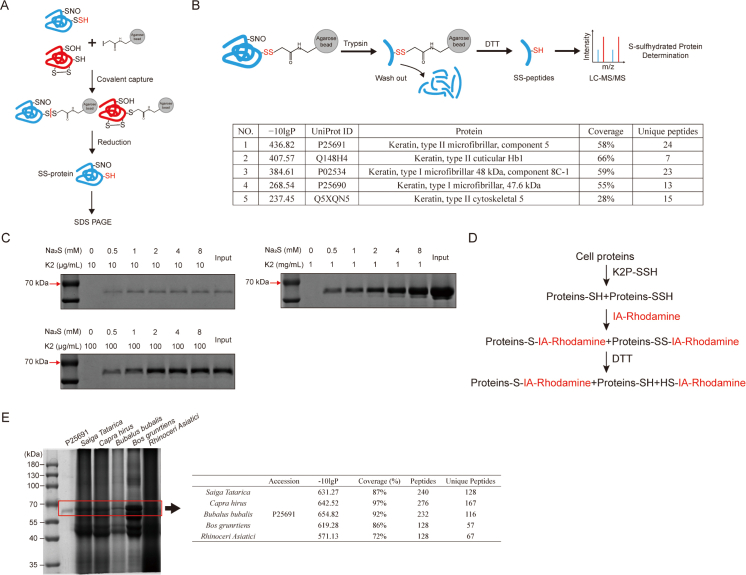
**K2-SSH regulates cellular protein sulfhydration levels.** (A) Schematic illustration of the RCCP workflow used for enrichment and isolation of putatively sulfhydrated proteins. (B) Summary of putatively sulfhydrated proteins identified by RCCP-based enrichment followed by nano-LC-MS/MS proteomic analysis. (C) SDS-PAGE analysis of K2 sulfhydration using the RCCP method following treatment with the indicated concentrations of Na_2_S for 2 h (n = 3). (D) Schematic illustration of the workflow used for detection of protein sulfhydration using IA-Rhodamine labeling. (E) SDS-PAGE analysis of keratin proteins derived from various keratinous animal tissues, followed by in-gel digestion and nano-LC-MS/MS-based identification of K2.

H_2_S exerts concentration-dependent regulatory effects during inflammation, and excessive H_2_S accumulation may contribute to inflammatory and metabolic disturbances under pathological conditions [41,42]. In the present study, KF exhibited the capacity to modulate H_2_S homeostasis in inflammatory models. Together with our previous findings and earlier reports [43], these observations suggest that the -S-S- moiety of KF may react with excessive H_2_S to generate -SSH groups, thereby helping maintain H_2_S homeostasis during inflammatory stress. Accordingly, we employed the RCCP method [29] to enrich protein sulfhydration signals (Fig. 1E–S1A). Given the methodological limitations of RCCP, proteins identified by this approach are referred to as putatively sulfhydrated proteins unless otherwise validated. Thiol-reactive agarose resin was used to capture thiol-containing proteins, followed by DTT-mediated elution to enrich putatively sulfhydrated proteins. The results showed that RCCP-enriched putatively sulfhydrated proteins were primarily detected in samples co-incubated with microbiota supernatant and KF. In contrast, pre-blocking KF with IAA appeared to reduce the enrichment signals (Fig. 1E). Furthermore, time-course analysis demonstrated that KF sulfhydration reached a peak at 2 h (Fig. 1F). Based on these findings, RCCP enrichment combined with MS-based identification was further performed, leading to the identification of 97 putatively sulfhydrated peptides (Fig. S1B, Table S3). Among these, K2 exhibited the highest −10lgP value and the greatest number of unique peptides (Fig. S1B). These findings suggest that KF consumes H_2_S via sulfhydration, thereby contributing to the maintenance of H_2_S homeostasis (Fig. 1A).

Previous studies [43,44] have demonstrated that RSS, including H_2_S and CysSSH, can mediate protein sulfhydration through multiple pathways (Fig. 1G). Among these, the oxidation of protein cysteine residues by ROS to form -SOH intermediates, followed by their reaction with RSS to generate -SSH, represents one of the most prevalent pathways [45,46]. To test whether sulfhydrated KF (KF-SSH) participates in such regulation, we further chemically synthesized KF-SSH and assessed intracellular sulfenylation and putatively sulfhydrated proteins using the DCP-Bio1 probe [30] and the RCCP assay, respectively. The results showed that, in bEnd.3 cells subjected to H_2_O_2_-induced oxidative stress or TNF-α-induced inflammation, KF-SSH significantly increased intracellular putatively sulfhydrated proteins compared with the model group and tended to reduce intracellular sulfenylation. These findings suggest that KF-SSH may shift the cellular cysteine redox modification profile from excessive sulfenylation toward sulfhydration, potentially limiting further oxidative modifications [31] (Fig. 1H and I). In summary, KF modulates H_2_S homeostasis, whereas KF-SSH regulates the cellular redox state and contributes to maintenance of cellular redox balance.

### 3.2 K2 is the most hyperreactive thiol-containing protein identified in KF

Hyperreactive -SH groups often serve as critical sites for PTM and enzymatic active centers in proteins, playing essential roles in various biological processes [33]. Consistent with this notion, our findings suggest that KF modulates H_2_S homeostasis and protein redox state through its -SH and -S-S- moieties. The reactivity of these moieties, particularly their conversion to -SSH, is closely linked to their biological function. Using a modified QTRP-based dimethyl labeling strategy, we profiled the reactivity of all -SH groups in KF and KF-SSH (Fig. 2A). The number of labeled peptides detected with the 100 μM probe was higher than that obtained with the 10 μM probe. (Fig. 2B). The criteria for peptide selection were defined as follows: intensity ≥ e8, and a mass spectrometry response ratio between peptides labeled with 100 μM and 10 μM probes ≤ 2. Peptides consistently identified across all three replicates were classified as hyperreactive peptides (Fig. 2C). A total of 17 hyperreactive -SH-containing peptides were identified in the KF group, and 7 in the KF-SSH group (Table S4). Notably, the precursor exhibiting the highest proportion of hyperreactive -SH peptides in both KF and KF-SSH was consistently identified as the K2 (Fig. 2C). We performed a Venn diagram analysis of three peptide sets: (i) the 97 sulfhydrated peptides identified from KF co-incubated with microbiota supernatant; (ii) the 17 hyperreactive peptides from KF; and (iii) the 7 hyperreactive peptides from KF-SSH. This analysis identified five overlapping peptides—four from K2 and one from Q148H4 (Fig. 2D). These findings indicate that K2 harbors the greatest number of hyperreactive -SH groups among KF. RCCP-based enrichment of putatively sulfhydrated proteins revealed that K2 undergoes dose-dependent sulfhydration upon Na_2_S treatment (Fig. 2E–S1C). The H_2_S-consuming capacity of K2 was evaluated using the lead acetate method, which showed a tendency for K2 to reduce H_2_S levels (Fig. 2F). Moreover, chemically synthesized K2-SSH tended to increase H_2_S levels (Fig. 2G).

As one of the key active sulfur species, we reason that the -SSH generated from K2-SSH exerts its biological effects by modulating intracellular -SSH and H_2_S levels. To investigate this, we employed the fluorescent probes SSP4 [47] and WSP-5 [45] to measure -SSH and H_2_S levels in RAW264.7 cells, respectively. K2P-SSH treatment significantly increased intracellular -SSH levels compared with the model group. Meanwhile, WSP-5 analysis showed a trend toward increased intracellular H_2_S levels under both basal and inflammatory conditions in LPS-treated cells (Fig. 2H). Fluorescence-PAGE [12,15] analysis of cellular protein sulfhydration similarly suggested a dose-dependent increase with K2-SSH treatment, peaking at 1 h (Fig. 2I–S1D). Collectively, these observations support a role for the hyperreactive -SH in K2 in modulating H_2_S levels through sulfhydration and influencing the redox state of cellular proteins, thereby potentially contributing to protection against oxidative stress.

### 3.3 K2 suppresses LPS-induced inflammatory fever *in vivo*

**Fig. 3:**
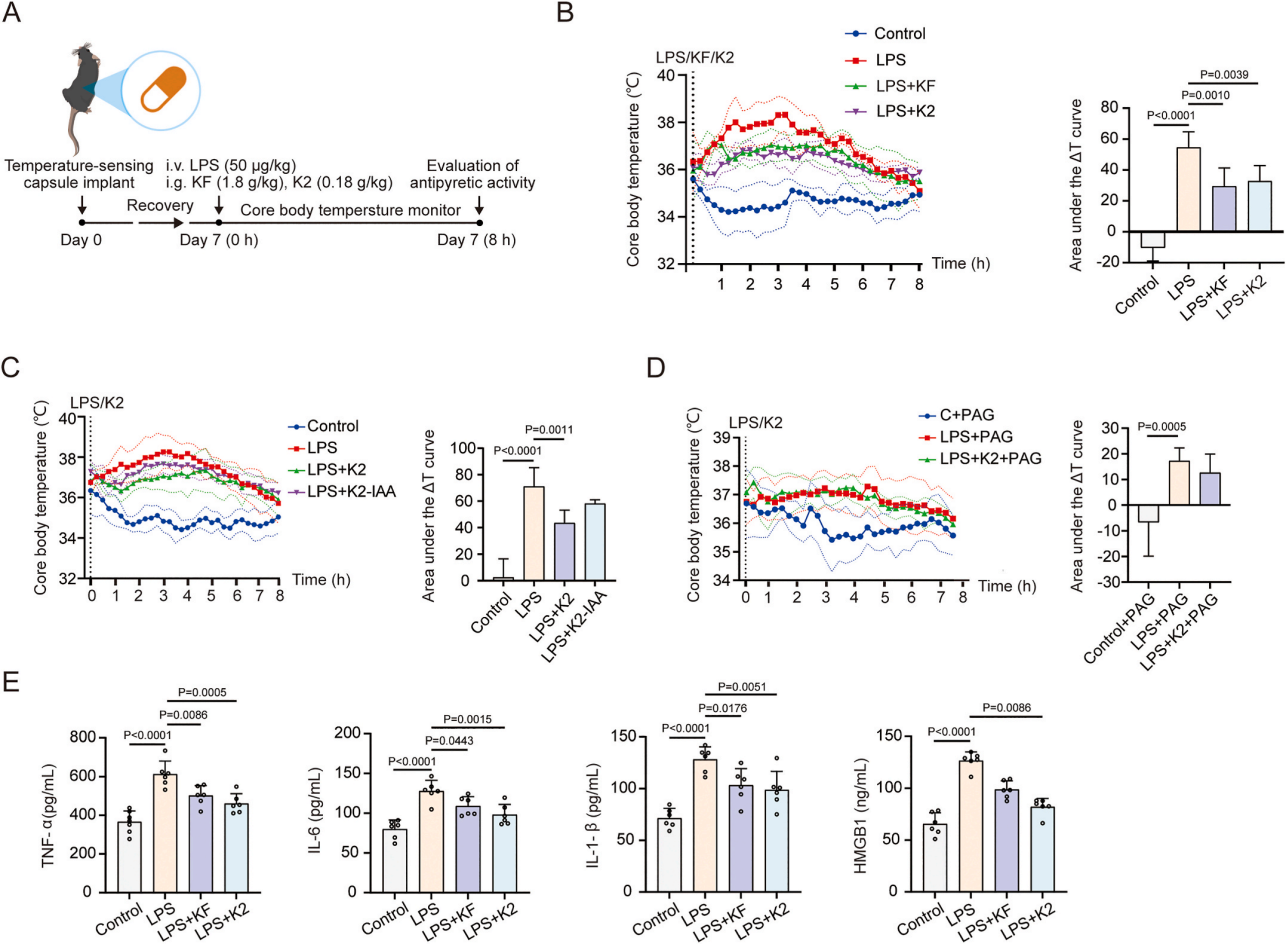
**Antipyretic and anti-inflammatory effects of K2.** (A) Schematic illustration of the remote thermometry protocol used for continuous monitoring of core body temperature in mice. (B−D) Time-course analysis of core body temperature from 0 to 8 h following LPS injection (50 μg/kg). KF (1.8 g/kg) and K2 (0.18 g/kg) were orally administered, whereas PAG (64 mg/kg) was injected for 3 days (n = 6). (E) Quantification of TNF-α, IL-6, IL-1β, and HMGB1 levels in mouse serum collected 5 h after KF (1.8 g/kg) and K2 (0.18 g/kg) administration using ELISA assays (n = 6). Data are presented as mean ± SD. One-way ANOVA followed by Dunnett's multiple-comparison was performed for **B, C, E**. Kruskal–Wallis test followed by Dunn's multiple comparisons was performed for **D,** and for HMGB1 levels in **E**.

KF-derived K2 modulates protein redox status and protects against excessive oxidation. As LPS-induced febrile mice exhibit marked oxidative-inflammatory stress, this model was selected to evaluate the antipyretic activity of K2. Oral administration of K2 and KF led to a reduction in body temperature within 30–60 min, an effect that persisted for approximately 3 h. Analysis of the AUC from 1 to 5 h post-administration across all experimental groups revealed a significantly reduced AUC in the K2 and KF treated group, indicating a potent and sustained antipyretic effect (Fig. 3B, S2A). To investigate the functional roles of the abundant -SH and -S-S- moieties in K2, we reduced the -S-S- moieties with DTT and blocked the -SH groups with IAA to generate K2-IAA. When administered at equivalent doses, K2-IAA exhibited no significant antipyretic effect, suggesting that the -SH groups are essential for the antipyretic activity of K2 (Fig. 3C). According to previous reports [12,45], CSE is a key enzyme involved in endogenous sulfur metabolism. To further explore the mechanistic involvement of this pathway, we pretreated mice with propargylglycine (PAG), a specific CSE inhibitor, to block H_2_S production and associated sulfhydration. Because PAG itself attenuated the LPS-induced fever [48], the results shown in Fig. 3D should be interpreted independently from those in Fig. 3B and C. Under these conditions, K2 failed to produce a significant antipyretic effect (Fig. 3D), suggesting that systemic sulfur metabolism may contribute to the biological effects of K2. ELISA results also showed that both K2 and KF reduced plasma levels of IL-1β, IL-6 and TNF-α. K2 also significantly reduced HMGB1 levels, whereas KF showed a trend toward lower HMGB1 levels (Fig. 3E). In summary, K2 exhibits antipyretic activity, and is a representative keratin found in keratin-based medicinal materials (Fig. S1E).

**Supplementary Fig. 2:**
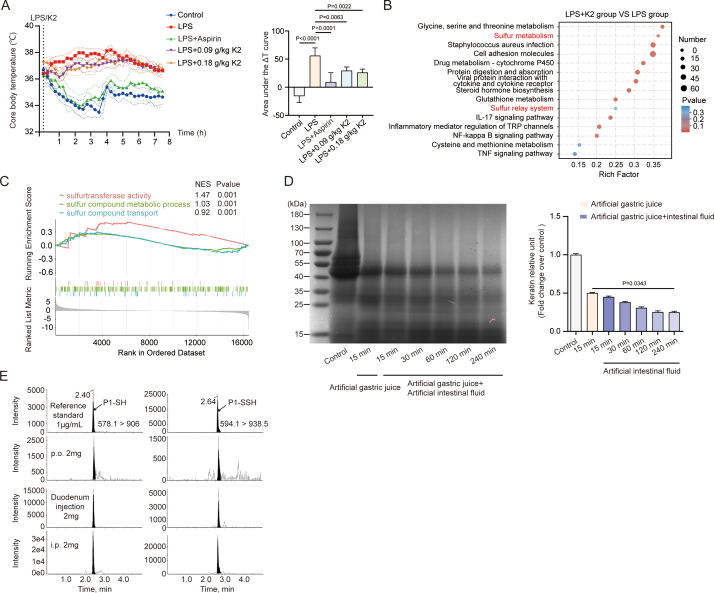
**Antipyretic effects and *in vivo* processing of K2.** (A) Antipyretic effect of K2 at the indicated doses in LPS-induced febrile mice (n = 5). (B, C) KEGG and GSEA enrichment analyses of the RNA-seq datasets from the LPS (50 μg/kg) group and K2-treated group (0.18 g/kg) (n = 6). GSEA identified significantly enriched pathways and corresponding normalized enrichment scores (NES) (p < 0.001). (D) Simulated gastrointestinal digestion of KF in artificial gastric fluid and artificial intestinal fluid at the indicated time points (n = 3). (E) Detection of P1-SH and P1-SSH in the mouse serum collected 30 min after administration by LC-MS/MS analysis. Data are presented as mean ± SD. One-way ANOVA followed by Dunnett's multiple-comparison was performed for **A**. Kruskal–Wallis test followed by Dunn's multiple comparisons was performed for **D.**

We next explored the sulfur metabolism pathways involved in K2 and its potential mechanistic effects. Transcriptomic sequencing of intestinal tissue was performed to analyze mRNA expression changes in febrile mice following K2 administration. The results showed that, compared with the model group, K2 intervention resulted in 2551 differentially expressed genes in the small intestine, comprising 1135 upregulated genes and 1416 downregulated genes. These differentially expressed genes were enriched in multiple sulfur-related pathways, including sulfur metabolism, the sulfur relay system, sulfur compound transport, sulfur compound metabolic processes, and sulfur transferase activity (Fig. S2B and S2C). These findings provide preliminary evidence that, within the mouse intestinal environment, K2 exerts its antipyretic effect through the participation of its -SH and -S-S- moieties in sulfur transport.

### 3.4 K2P-SSH exhibits enhanced anti-inflammatory and antioxidant activity

**Fig. 4:**
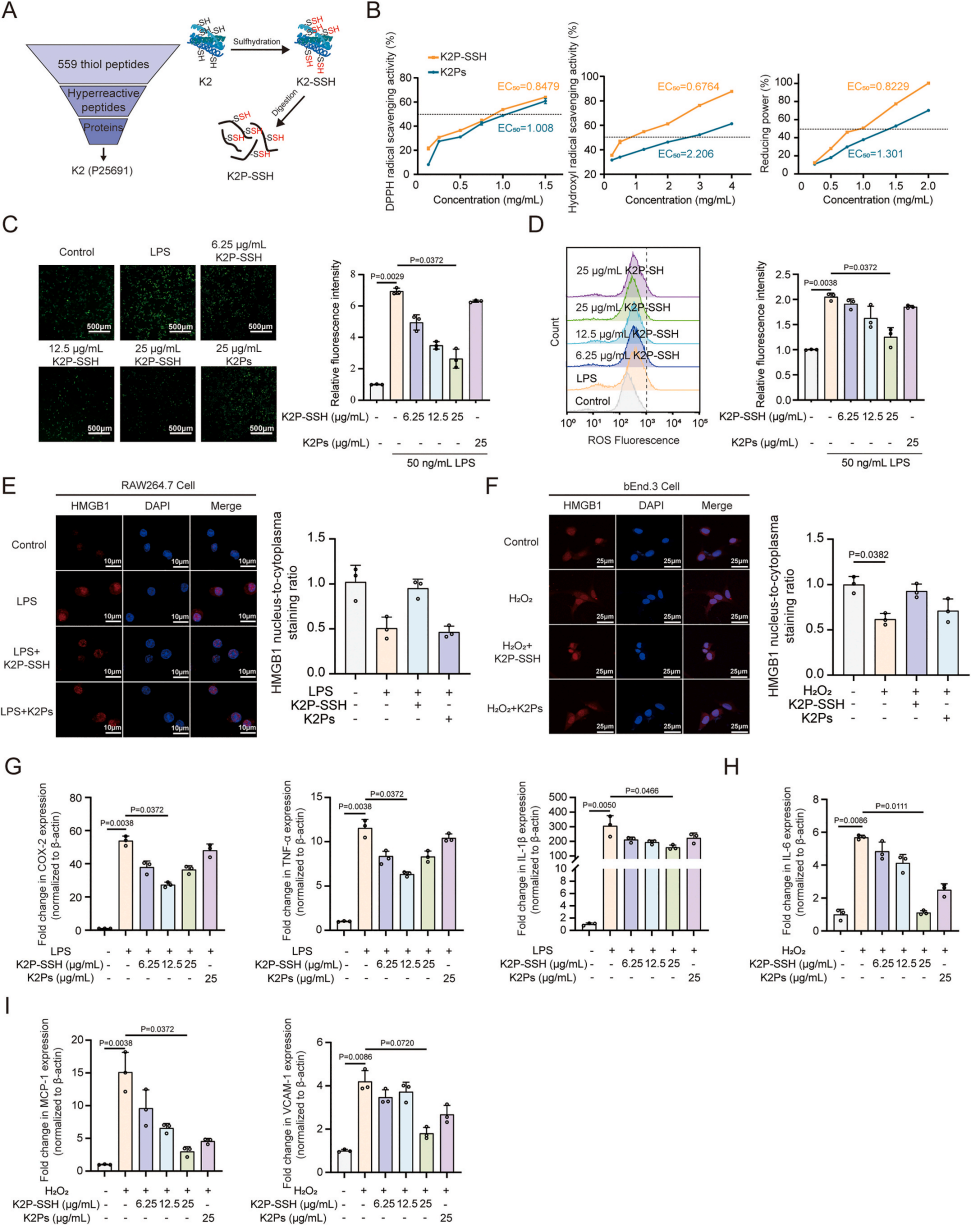
**K2P-SSH shows superior anti-inflammatory and antioxidant activity compared with K2Ps.** (A) Schematic illustration of the discovery and preparation of K2P-SSH. (B) Comparison of the DPPH radical scavenging activity, hydroxyl radical scavenging activity, and reducing power of K2Ps and K2P-SSH at different concentrations. EC_50_ values are indicated in the corresponding panels (n = 3). (C, D) Representative fluorescence images and flow cytometry analysis of intracellular ROS levels in RAW264.7 cells pretreated with K2P-SSH or K2Ps for 12 h, followed by LPS stimulation for 4 h (n = 3). (E, F) IF analysis of HMGB1 in RAW264.7 and bEnd.3 cells pretreated with K2P-SSH (25 μg/mL) and K2Ps (25 μg/mL) for 12 h, followed by LPS stimulation for 4 h (n = 3, Scale bar = 10 μm and 25 μm, respectively). (G, H, I) mRNA expression levels of pro-inflammatory cytokines, including IL-6, MCP-1, VCAM-1, COX-2, TNF-α and IL-1β, determined by qRT-PCR (n = 3). Data are presented as mean ± SD. Mann–Whitney *U* test was performed for **B**. Kruskal–Wallis test followed by Dunn's multiple comparisons was performed for **C, D, G−I.**

Through screening 559 KF-derived peptides for thiol reactivity, we identified K2 as harbouring the greatest number of hyperreactive -SH groups. Further investigation of KF processing in the simulated intestinal environment revealed that it rapidly undergoes sulfhydration (Fig. 1F), with degradation within 15–30 min of exposure [49] (Fig. 4A, S2D). To determine whether the hyperreactive peptides derived from digested K2 can be systemically absorbed, we synthesized P1 peptide (TFSSCSAVAPK) containing a hyperreactive -SH group. Following oral, duodenal, and intraperitoneal administration, both the -SH and -SSH forms of P1 could be detected in serum, suggesting that K2 may undergo digestive processing and be absorbed predominantly in these peptide forms (Fig. S2E). Subsequently, we generated K2Ps through simulated intestinal fluid digestion of the K2 protein and then synthesized K2P-SSH. The characterization data are summarized in Table S5.

**Supplementary Fig. 3:**
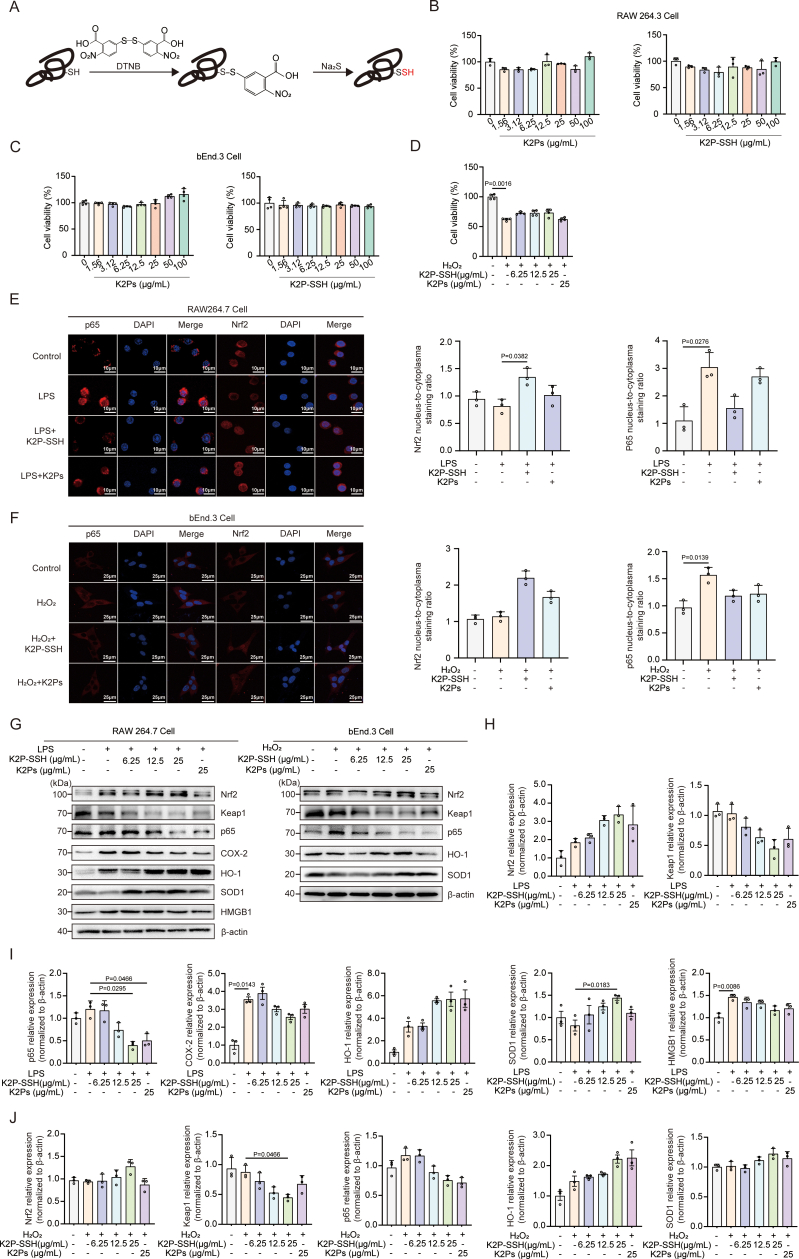
**K2P-SSH exhibits anti-inflammatory and antioxidative activity through the NF-κB/Nrf2/HMGB1 signaling axis.** (A) Schematic illustration of K2P-SSH synthesis. (B, C) Cell viability of RAW264.7 and bEnd.3 cells following treatment with K2Ps or K2P-SSH at the indicated concentrations for 24 h (n = 3). (D) Cell viability of bEnd.3 cells treated with H_2_O_2_ (1.1 mM) alone or in combination with K2P-SSH or K2Ps at the indicated concentrations (n = 3). (E, F) IF analysis of p65 and Nrf2 levels in RAW264.7 and bEnd.3 cells pretreated with K2P-SSH (25 μg/mL) or K2Ps (25 μg/mL) for 12 h, followed by LPS or H_2_O_2_ stimulation for 4 h (n = 3, Scale bar: 25 μm). (G−J) Western blot analysis of Nrf2, Keap1, p65, COX-2, HO-1, SOD1, and HMGB1 protein levels in RAW264.7 and bEnd.3 cells pretreated with K2P-SSH or K2Ps for 12 h, followed by LPS or H_2_O_2_ stimulation for 4 h (n = 3). Data are presented as mean ± SD. Kruskal–Wallis test followed by Dunn's multiple comparisons was performed for **B−F, G−J.**

We systematically evaluated the *in vitro* anti-inflammatory and antioxidant activities of K2Ps and K2P-SSH. As shown in Fig. 4B, K2P-SSH exhibited greater radical scavenging and reducing capacities than K2Ps. The effects of K2Ps and K2P-SSH were assessed using an LPS-induced inflammatory model in RAW264.7 cells and an H_2_O_2_-induced oxidative stress model in bEnd.3 cells. Based on Cell Counting Kit-8 (CCK-8) cell viability assays, concentrations of 6.25, 12.5, and 25 μg/mL were selected as the working concentrations for K2P-SSH (Fig. S3B and S3C). K2P-SSH exhibited a trend toward protection against H_2_O_2_-induced cytotoxicity in bEnd.3 cells, with a more pronounced effect than K2Ps at the same concentration (Fig. S3D). Both ROS fluorescent probing and flow cytometry analyses indicated that K2P-SSH reduced intracellular ROS levels in LPS-stimulated RAW264.7 cells. Notably, 25 μg/mL K2P-SSH significantly reduced ROS levels compared with the model group (Fig. 4C and D).

Our previous studies have demonstrated that keratin peptides exert protective effects against oxidative-inflammatory stress by modulating the Nrf2/Hmox-1/HMGB1 and NF-κB signaling pathways [1]. Therefore, in LPS-stimulated RAW264.7 cells and H_2_O_2_-stressed bEnd.3 cells, IF assays were used to assess the nuclear-to-cytoplasmic translocation of HMGB1, p65, and Nrf2. LPS and H_2_O_2_ were associated with a trend toward increased HMGB1 nuclear export, whereas K2P-SSH tended to attenuate this translocation (Fig. 4E and F). Similarly, nuclear accumulation of p65 showed a trend toward increase upon LPS or H_2_O_2_ treatment, while K2Ps and K2P-SSH tended to reduce p65 nuclear localization (Fig. S3E and S3F). K2P-SSH significantly increased Nrf2 nuclear localization in RAW264.7 cells and tended to enhance Nrf2 nuclear accumulation in bEnd.3 cells compared with the model group (Fig. S3E and S3F).

qRT-PCR analysis showed that K2P-SSH significantly suppressed the levels of COX-2, IL-1β, and TNF-α in RAW264.7 cells. In bEnd.3 cells, K2P-SSH significantly reduced the levels of IL-6 and monocyte chemoattractant protein-1 (MCP-1), while vascular cell adhesion molecule-1 (VCAM-1) levels tended to decrease following K2P-SSH treatment. In contrast, K2Ps tended to reduce the levels of IL-6, MCP-1, and VCAM-1 in bEnd.3 cells (Fig. 4G, H, 4I). The Western blot results indicated that, in RAW264.7 (inflammatory models) and bEnd.3 (oxidative stress models) cells, K2P-SSH treatment tended to upregulate Nrf2, HO-1, and SOD1, while Keap1 and p65 tended to be downregulated in a dose-dependent manner. In RAW264.7 cells, p65 and SOD1 levels were significantly altered following K2P-SSH treatment, whereas COX-2 and HMGB1 exhibited a trend toward reduction. In bEnd.3 cells, only Keap1 protein levels showed significant changes (Fig. S3G−J). Overall, these results suggest that K2P-SSH modulates the NF-κB/Nrf2/HMGB1 signaling axis and contributes to enhanced anti-inflammatory and antioxidant responses compared with K2Ps.

### 3.5 K2P-SSH induces HMGB1 sulfhydration and attenuates oxidative-inflammatory stress

**Fig. 5:**
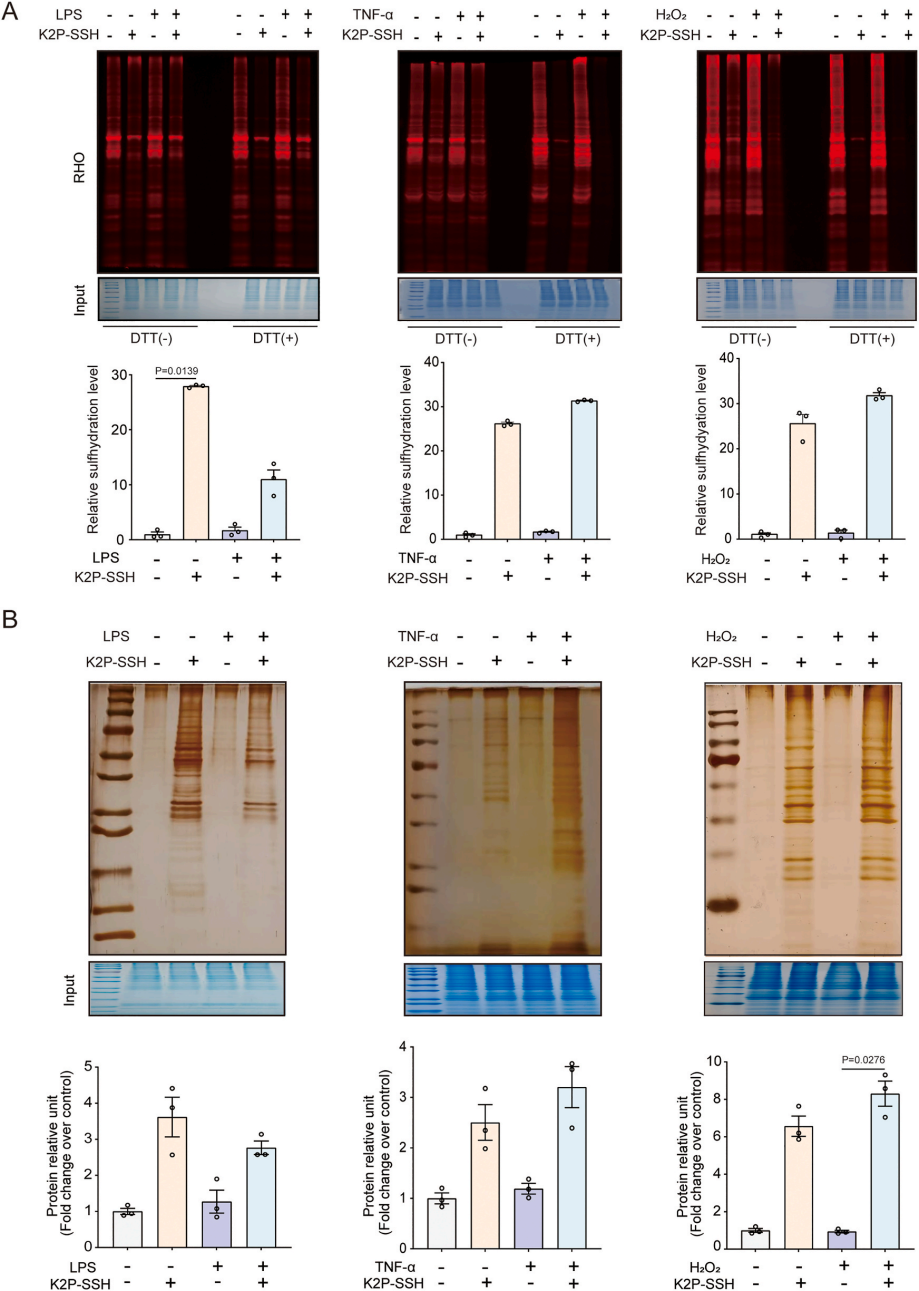
**K2P-SSH induces widespread protein sulfhydration in bEnd.3 and RAW264.7 cells.** (A) Detection of sulfhydrated proteins in RAW264.7 cells using the IA-Rhodamine probe. Cells were stimulated with LPS, H_2_O_2_ or TNF-α for 4 h, followed by treatment with K2-SSH (1 mg/mL) for 1 h. Cells were subsequently lysed and subjected to IA-Rhodamine labeling. $\text{Level}\;\text{of}\;\text{sulfhydration}=\frac{\text{Rhodamine}\;\text{intensity}\;\left(\text{Before}\;\text{DTT}\right)-\text{Rhodamine}\;\text{intensity}\;\left(\text{After}\;\text{DTT}\right)}{\text{Rhodamine}\;\text{intensity}\;\left(\text{Before}\;\text{DTT}\right)}$ (n = 3). (B) RCCP-based enrichment analysis of putatively sulfhydrated proteins in RAW264.7 cells. Cells were stimulated with LPS, H_2_O_2_, or TNF-α for 4 h, followed by K2P-SSH (1 mg/mL) treatment for 1 h. Cells were subsequently lysed and subjected to RCCP-based enrichment using SulfoLink Coupling Agarose Resin (n = 3). Data are presented as mean ± SD. Kruskal–Wallis test followed by Dunn's multiple comparisons was performed for **A, B.**

**Fig. 6:**
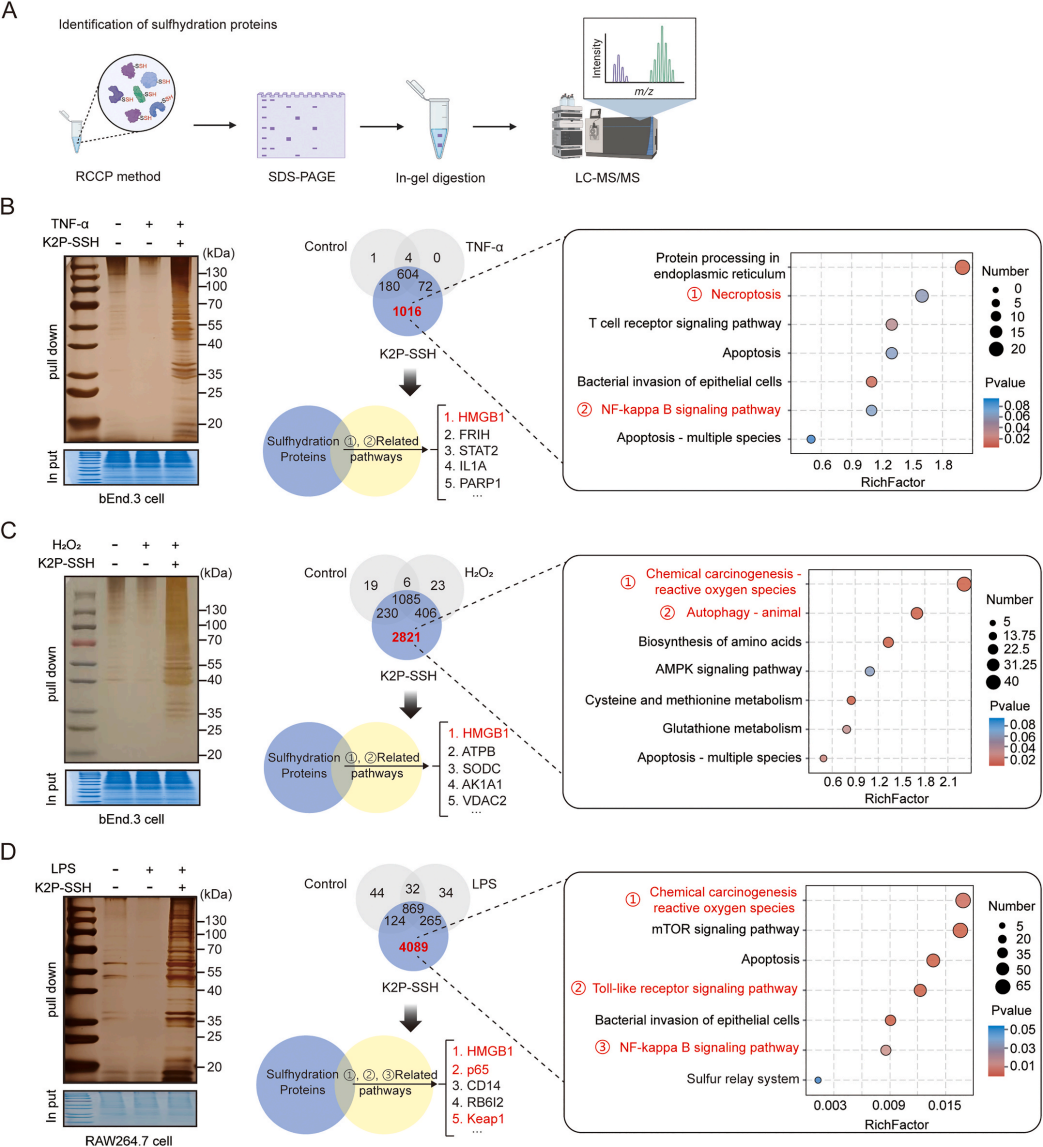
**Identification of HMGB1, Keap1, and p65 as major putatively sulfhydrated targets of K2P-SSH in bEnd.3 and RAW264.7 cells.** (A) Schematic illustration of the workflow for RCCP-based enrichment and in-gel digestion used for identification of putatively sulfhydrated proteins. (B−D) Putatively sulfhydrated proteins were identified in H_2_O_2_-induced bEnd.3 cells, TNF-α-induced bEnd.3 cells, and LPS-induced RAW264.7 cells following K2P-SSH (1 mg/mL) treatment for 1 h. Cells were subsequently lysed, and putatively sulfhydrated proteins were enriched using the RCCP method, followed by in-gel digestion and nano-LC-MS/MS-based protein identification. Venn diagram analysis was performed to compare differentially enriched putatively sulfhydrated proteins among groups, followed by KEGG pathway enrichment analysis to identify potential sulfhydration-regulated signaling pathways and target proteins.

**Supplementary Fig. 4:**
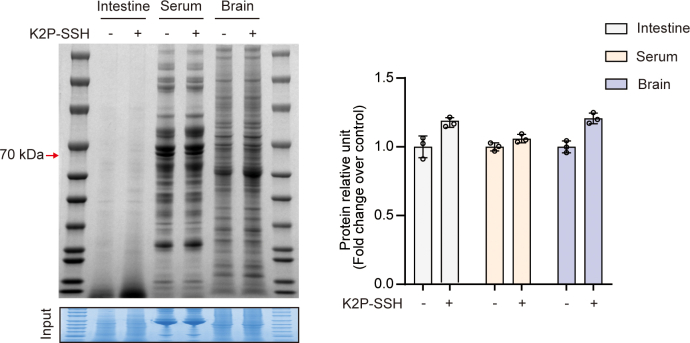
**K2P-SSH induces protein sulfhydration *in vivo***. RCCP-based analysis of protein sulfhydration levels in mouse intestine, serum, and brain tissues collected 2 h after oral administration of K2P-SSH (0.18 g/kg) (n = 3). Data are presented as mean ± SD. Mann–Whitney *U* test was performed.

To further investigate the role of K2P-SSH in protein sulfhydration, this study utilized Fluorescence-PAGE and RCCP to evaluate intracellular putatively sulfhydrated proteins. K2P-SSH tended to increase protein sulfhydration-associated signals across three distinct cellular models—the LPS-induced inflammatory model in RAW264.7 cells, the TNF-α-induced inflammatory model in bEnd.3 cells, and the H_2_O_2_-induced oxidative stress model in bEnd.3 cells—under both basal and stimulated conditions. These observations are consistent with a potential role for K2P-SSH in limiting oxidative-inflammatory stress-associated protein damage (Fig. 5A and B). To further assess whether this effect could also be observed *in vivo*, mice were orally administered K2P-SSH and protein sulfhydration levels were evaluated in intestinal tissue, plasma, and brain tissue. A trend toward increased protein sulfhydration-associated signals was observed in intestinal and brain tissues following K2P-SSH treatment compared with the control group (Fig. S4). Moreover, by combining the RCCP method with in-gel digestion experiments, putatively sulfhydrated proteins induced by K2P-SSH in the three cell models were successfully identified (Fig. 6A). Venn diagram analysis and KEGG pathway enrichment identified HMGB1 as the most consistently enriched putatively sulfhydrated proteins across all models, while p65 and Keap1 were additionally identified in LPS-stimulated RAW264.7 cells. (Fig. 6B–D, Table S6). Collectively, these findings suggest that K2P-SSH modulates protein sulfhydration-associated redox signaling, which may contribute to its anti-inflammatory and antioxidant effects.

**Fig. 7:**
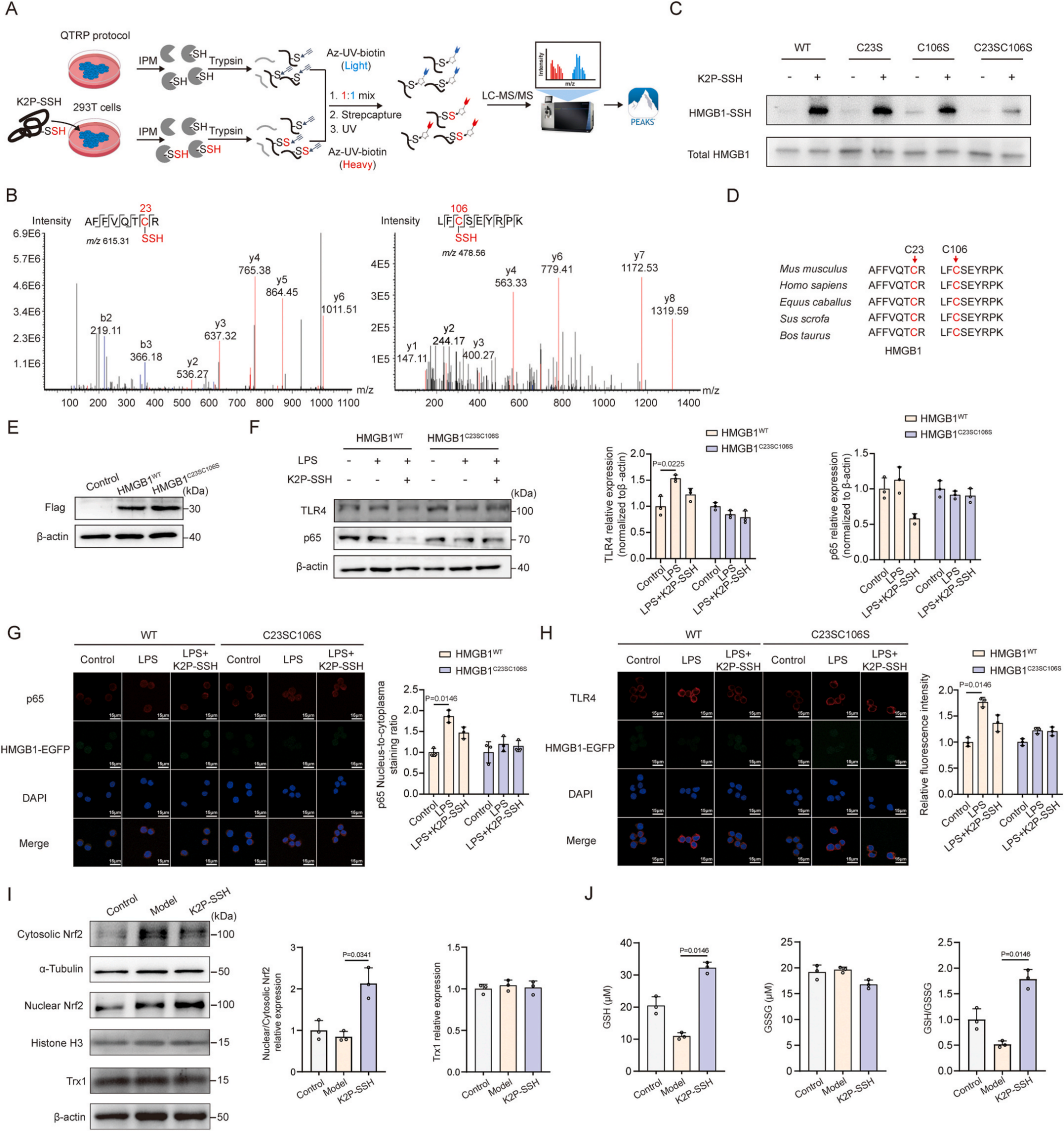
**Sulfhydration of HMGB1 contributes to the anti-inflammatory and antioxidative activities of K2P-SSH.** (A) Schematic illustration of the workflow used for identification of sulfhydrated proteins in 293 T cells using the QTRP method following treatment with K2P-SSH (1 mg/mL) for 1 h. (B) Representative MS/MS spectra of HMGB1 peptides containing sulfhydrated Cys23 and Cys106 identified in 293 T cells. (C) Western blot analysis of HMGB1 sulfhydration levels in RAW264.7 cells expressing wild-type HMGB1 or the HMGB1^C23SC106S^ mutant. Cells were treated with K2P-SSH (1 mg/mL) for 1 h, followed by cell lysis and RCCP-based enrichment. (D) Chart displaying the conserved nature of Cys23 and Cys106 residues in HMGB1 among mammals. (E) Western blot validation of FLAG-tagged HMGB1^C23SC106S^ mutant levels in RAW264.7 cells. (F) Western blot analysis of TLR4 and p65 levels in the HMGB1^WT^ and HMGB1^C23SC106S^ cells pretreated with K2P-SSH (25 μg/mL) for 12 h, followed by LPS stimulation for 4 h (n = 3). (G) IF analysis of p65 levels in the HMGB1^WT^ and HMGB1^C23SC106S^ cells pretreated with K2P-SSH (25 μg/mL) for 12 h, followed by LPS stimulation for 4 h (n = 3, Scale bar: 15 μm). (H) IF analysis of TLR4 levels in the HMGB1^WT^ and HMGB1^C23SC106S^ cells pretreated with K2P-SSH (25 μg/mL) for 12 h, followed by LPS stimulation for 4 h (n = 3, Scale bar: 15 μm). (I) Western blot analysis of cytosolic Nrf2, nuclear Nrf2, and Trx1 levels in RAW264.7 cells pretreated with K2P-SSH (25 μg/mL) for 12 h, followed by LPS stimulation for 4 h (n = 3). (J) Intracellular GSH levels, GSSG levels, and GSH/GSSG ratios in RAW264.7 cells pretreated with K2P-SSH (25 μg/mL) for 12 h, followed by LPS stimulation for 4 h (n = 3). Data are presented as mean ± SD. Kruskal–Wallis test followed by Dunn's multiple comparisons was performed for **F−J.**

To identify the specific cysteine site of HMGB1 undergoing sulfhydration, we employed the QTRP chemical proteomics approach to identify sulfhydration sites induced by K2P-SSH. As shown in Fig. 7A and B, HMGB1 was overexpressed in 293 T cells, which were then divided into a control group and a K2P-SSH treatment group. Proteins from the control and treatment groups were labeled with light and heavy isotopically labeled UV-cleavable biotin-azide reagents, respectively. Following analysis using Peaks software, Cys23 and Cys106 of HMGB1 were identified as carrying the IPM-tagged sulfhydration. To further identify the sulfhydration sites of HMGB1, we generated site-directed mutations at different cysteine residues of HMGB1 and evaluated the effects of K2P-SSH on HMGB1 sulfhydration using the RCCP assay. Following K2P-SSH treatment, increased sulfhydration-associated signals were observed in all HMGB1 mutants except the C23SC106S double mutant, which exhibited little change relative to its untreated control (Fig. 7C). Furthermore, sequence alignment showed that Cys23 and Cys106 are highly conserved across various mammals, providing additional support for their potential roles as sulfhydration sites (Fig. 7D). These observations support the possibility that Cys23 and Cys106 are major sites involved in K2P-SSH-induced HMGB1 sulfhydration. Therefore, the HMGB1^C23SC106S^ double mutant was selected for subsequent functional validation.

We constructed a FLAG-tagged HMGB1 site-directed mutant HMGB1^C23SC106S^ in RAW264.7 cells (Fig. 7E). LPS was used to establish an inflammatory model in both wild-type (WT) and mutant RAW264.7 cells to evaluate the effects of K2P-SSH. The results showed that, in HMGB1^WT^ cells, K2P-SSH tended to reduce the expression levels of TLR4 and p65 proteins, whereas these effects appeared attenuated in HMGB1^C23SC106S^ cells (Fig. 7F). IF analysis further showed that K2P-SSH tended to reduce LPS-induced p65 nuclear accumulation and TLR4 levels in HMGB1^WT^ cells. In contrast, these changes were less evident in HMGB1^C23SC106S^ cells (Fig. 7G and H). Collectively, these observations suggest that Cys23 and Cys106 participate in K2P-SSH-mediated regulation of HMGB1 signaling and contribute to the anti-inflammatory effects associated with K2P-SSH treatment.

**Supplementary Fig. 5:**
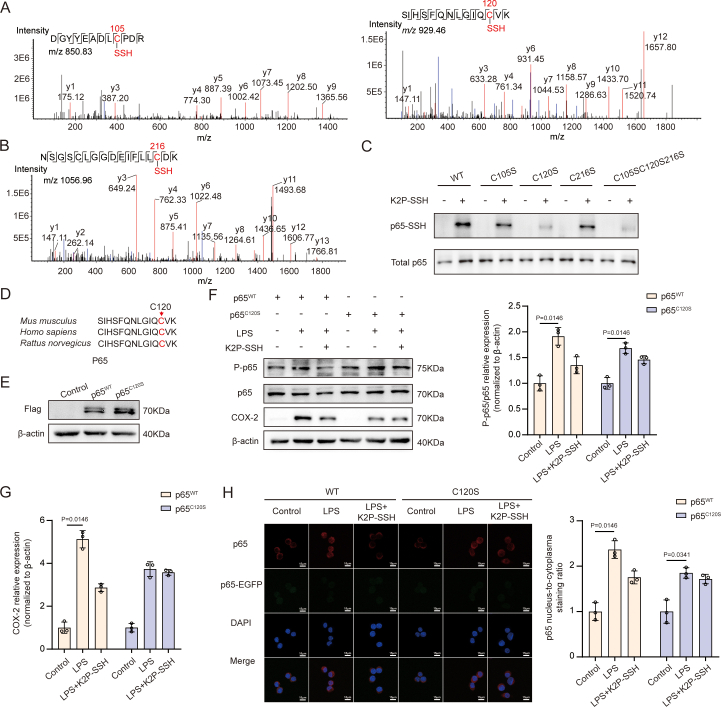
**Sulfhydration of p65 is essential for the anti-inflammatory and antioxidative activities of K2P-SSH.** (A, B) Representative MS/MS spectra of p65 peptides containing sulfhydrated Cys105, Cys120 and Cys216 identified in 293T cells. (C) Western blot analysis of p65 sulfhydration levels in RAW264.7 cells expressing wild-type or mutant p65. Cells were treated with K2P-SSH (1 mg/mL) for 1 h, followed by cell lysis and RCCP-based enrichment. (D) Chart displaying the conserved nature of Cys120 residues in p65 among mammals. (E) Western blot validation of FLAG-tagged p65^C120S^ mutant levels in RAW264.7 cells. (F, G) Western blot analysis of P-p65, p65 and COX-2 levels in p65^WT^ and p65^C120S^ cells pretreated with K2P-SSH (25 μg/mL) for 12 h, followed by LPS stimulation for 4 h (n = 3). (H) IF analysis of p65 levels in p65^WT^ and p65^C120S^ cells pretreated with K2P-SSH (25 μg/mL) for 12 h, followed by LPS stimulation for 4 h (n = 3, Scale bar: 15 μm). Data are presented as mean ± SD. Kruskal–Wallis test followed by Dunn's multiple comparisons was performed for **F−H.**

**Supplementary Fig. 6:**
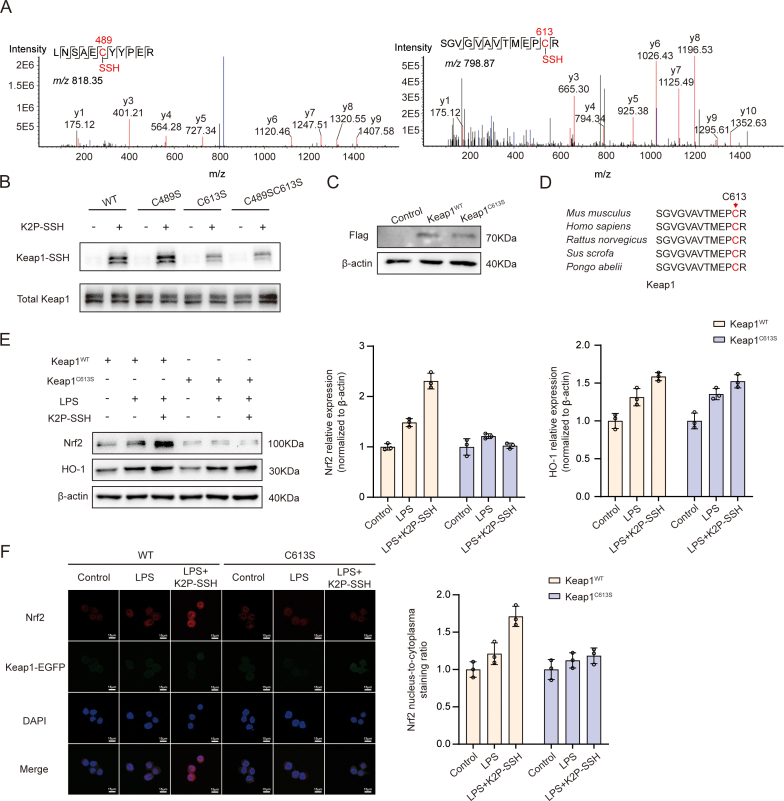
**Sulfhydration of Keap1 is essential for the anti-inflammatory and antioxidative activities of K2P-SSH.** (A) Representative MS/MS spectra of Keap1 peptides containing sulfhydrated Cys489 and Cys613 identified in 293T cells. (B) Western blot analysis of Keap1 sulfhydration levels in RAW264.7 cells expressing wild-type or mutant Keap1. Cells were treated with K2P-SSH (1 mg/mL) for 1 h, followed by cell lysis and RCCP-based enrichment. (C) Western blot validation of FLAG-tagged Keap1^C613S^ mutant levels in RAW264.7 cells. (D) Chart displaying the conserved nature of Cys613 residues in Keap1 among mammals. (E) Western blot analysis of Nrf2 and HO-1 levels in Keap1^WT^ and Keap1^C613S^ cells pretreated with K2P-SSH (25 μg/mL) for 12 h, followed by LPS stimulation for 4 h (n = 3). (F) IF analysis of Nrf2 levels in the Keap1^WT^ and Keap1^C613S^ cells pretreated with K2P-SSH (25 μg/mL) for 12 h, followed by LPS stimulation for 4 h (n = 3, Scale bar: 15 μm). Data are presented as mean ± SD. Kruskal–Wallis test followed by Dunn's multiple comparisons was performed for **E, F.**

Given that p65 and Keap1 were additionally identified as candidate putatively sulfhydrated proteins, we next investigated their sulfhydration sites and functional effects using a similar strategy. QTRP analysis identified Cys105, Cys120, and Cys216 of p65, as well as Cys489 and Cys613 of Keap1, as potential K2P-SSH-induced sulfhydration sites (Figs. S5A, S5B, S6A). To further validate these findings, individual p65 mutants (C105S, C120S, and C216S) and Keap1 mutants (C489S and C613S) were generated and analyzed using RCCP-based enrichment assays. Compared with the corresponding wild-type proteins, p65^C120S^ and Keap1^C613S^ mutants exhibited lower sulfhydration signals following K2P-SSH treatment (Figs. S5C and S6B). Given their high conservation among mammals, these residues were selected for subsequent functional validation (Figs. S5D and S6D). Functional studies showed that K2P-SSH tended to exert weaker effects on COX-2 levels (Fig. S5F and S5G) and p65 nuclear translocation (Fig. S5F and S5H) in p65^C120S^ cells. Likewise, K2P-SSH tended to show reduced effects on Nrf2 nuclear accumulation (Fig. S6E) and Nrf2/HO-1 levels in Keap1^C613S^ cells (Fig. S6F). These observations suggest that sulfhydration of p65^C120^ and Keap1^C613^ may contribute to the anti-inflammatory and antioxidant actions of K2P-SSH.

To further evaluate the downstream antioxidant responses associated with K2P-SSH treatment, Nrf2 nuclear translocation was assessed by nuclear/cytoplasmic fractionation followed by Western blot analysis. K2P-SSH significantly increased nuclear Nrf2 protein levels compared with the model group, supporting activation of the Nrf2 pathway (Fig. 7I). We further examined intracellular GSH/GSSG levels and Trx1 levels. LPS stimulation reduced intracellular GSH levels and the GSH/GSSG ratio, whereas K2P-SSH significantly restored both parameters. In contrast, no obvious changes in Trx1 levels were observed among the control, model, and K2P-SSH-treated groups (Fig. 7J). These findings suggest that K2P-SSH may partially modulate cellular redox homeostasis through regulation of the glutathione system, with no clear evidence for involvement of Trx1 upregulation.

### 3.6 K2P-SSH-induced sulfhydration of HMGB1 Cys23 and Cys106 is associated with reduced HMGB1–TLR4 interaction

HMGB1 perpetuates the oxidative-inflammatory stress state under pathological conditions. The redox status of its three conserved cysteines—Cys23, Cys45, and Cys106—critically dictates its structural and functional outcomes. Upon secretion, HMGB1 forms intramolecular (Cys23-Cys45, disulfide HMGB1) and intermolecular (Cys106-Cys106, dimerized HMGB1) disulfide bonds, generating redox-dependent conformations that promote HMGB1–TLR4 interaction and downstream inflammatory signaling [20,50]. Consistent with previous studies showing that the HMGB1^C106S^ mutation impairs HMGB1–TLR4 binding [51], the HMGB1^C23SC106S^ mutant described above exhibited minimal changes in TLR4 and p65 levels following K2P-SSH treatment. These findings suggest that Cys23 and Cys106 contribute to the redox-dependent regulation of HMGB1 function.

**Fig. 8:**
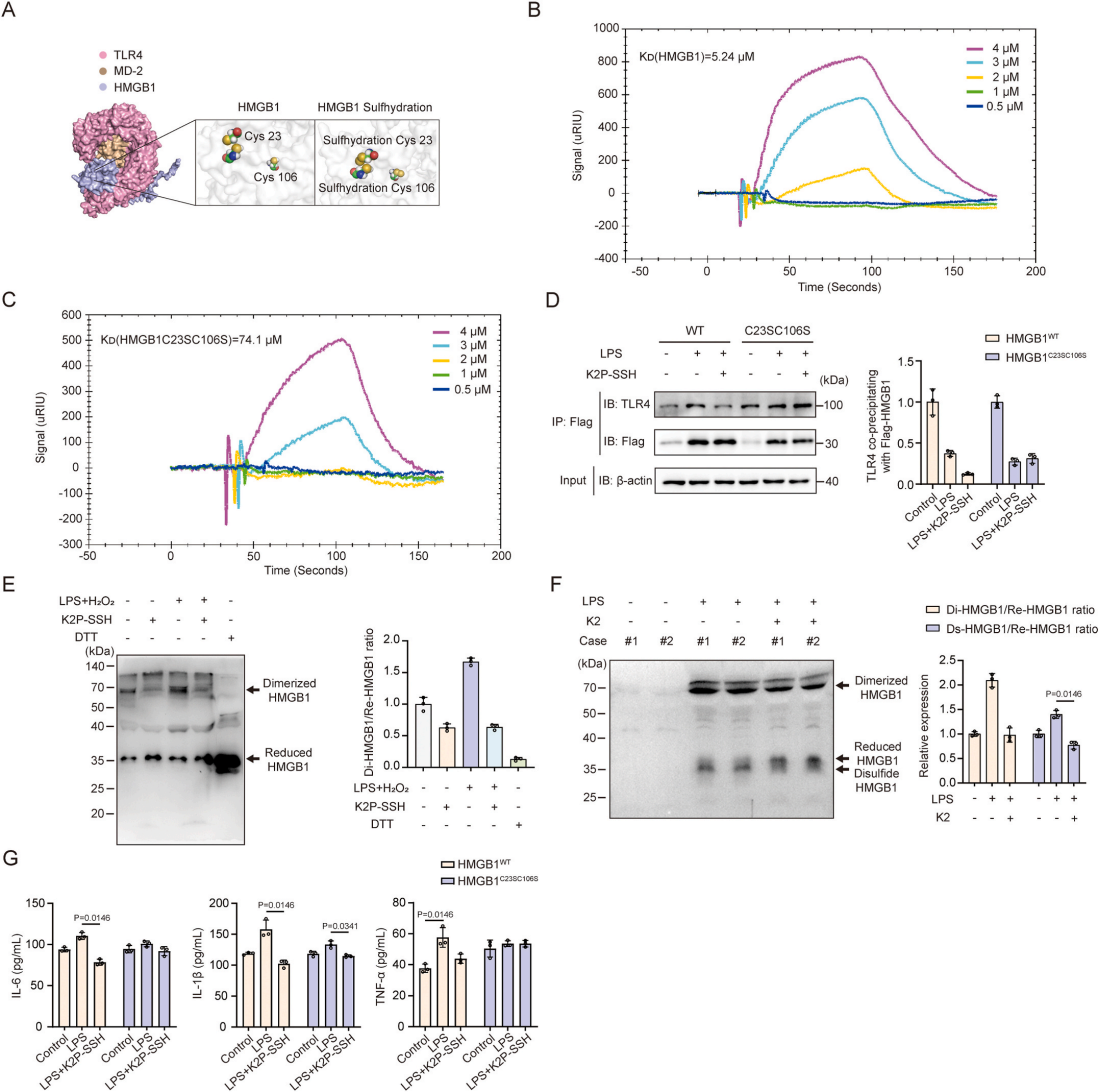
**Cys23 and Cys106 are required for the effect of K2P-SSH on HMGB1–TLR4 interaction.** (A) Molecular docking analysis showing that sulfhydration of HMGB1 alters the interaction pattern of the TLR4/MD-2/HMGB1 complex. (B, C) SPR analysis of HMGB1 binding to TLR4/MD2. HMGB1^WT^ and HMGB1^C23SC106S^ proteins were serially diluted starting from 4 μM and flowed over TLR4/MD2 immobilized on the sensor chip. (D) CO-IP analysis of HMGB1 interaction with TLR4 in cells expressing Flag-HMGB1^WT^ and Flag-HMGB1^C23SC106S^, pretreated with K2P-SSH (25 μg/mL) for 12 h, followed by LPS stimulation for 4 h. Protein interactions were detected by Western blot analysis (n = 3). (E) Reduced and dimerized HMGB1 (100 ng/μL) were incubated with LPS (50 ng/mL), H_2_O_2_ (50 μM) and K2P-SSH (25 μg/mL) in normal mouse serum for 45 min, followed by Western blot analysis of Re- and Ds-HMGB1 levels (n = 3). (F) Western blot analysis of disulfide HMGB1, dimerized HMGB1 and reduced HMGB1 protein forms in mouse serum. Reduced HMGB1 (*Re*-HMGB1), Disulfide HMGB1 (Ds-HMGB1) and Dimerized HMGB1 (Di-HMGB1) are indicated (n = 3). (G) ELISA analysis of TNF-α, IL-6, and IL-1β levels in the supernatants of HMGB1^WT^ and HMGB1^C23SC106S^ RAW264.7 cells pretreated with K2P-SSH (25 μg/mL) for 12 h, followed by LPS stimulation for 4 h (n = 3). Data are presented as mean ± SD. Kruskal–Wallis test followed by Dunn's multiple comparisons was performed for **D−G.**

**Fig. 9:**
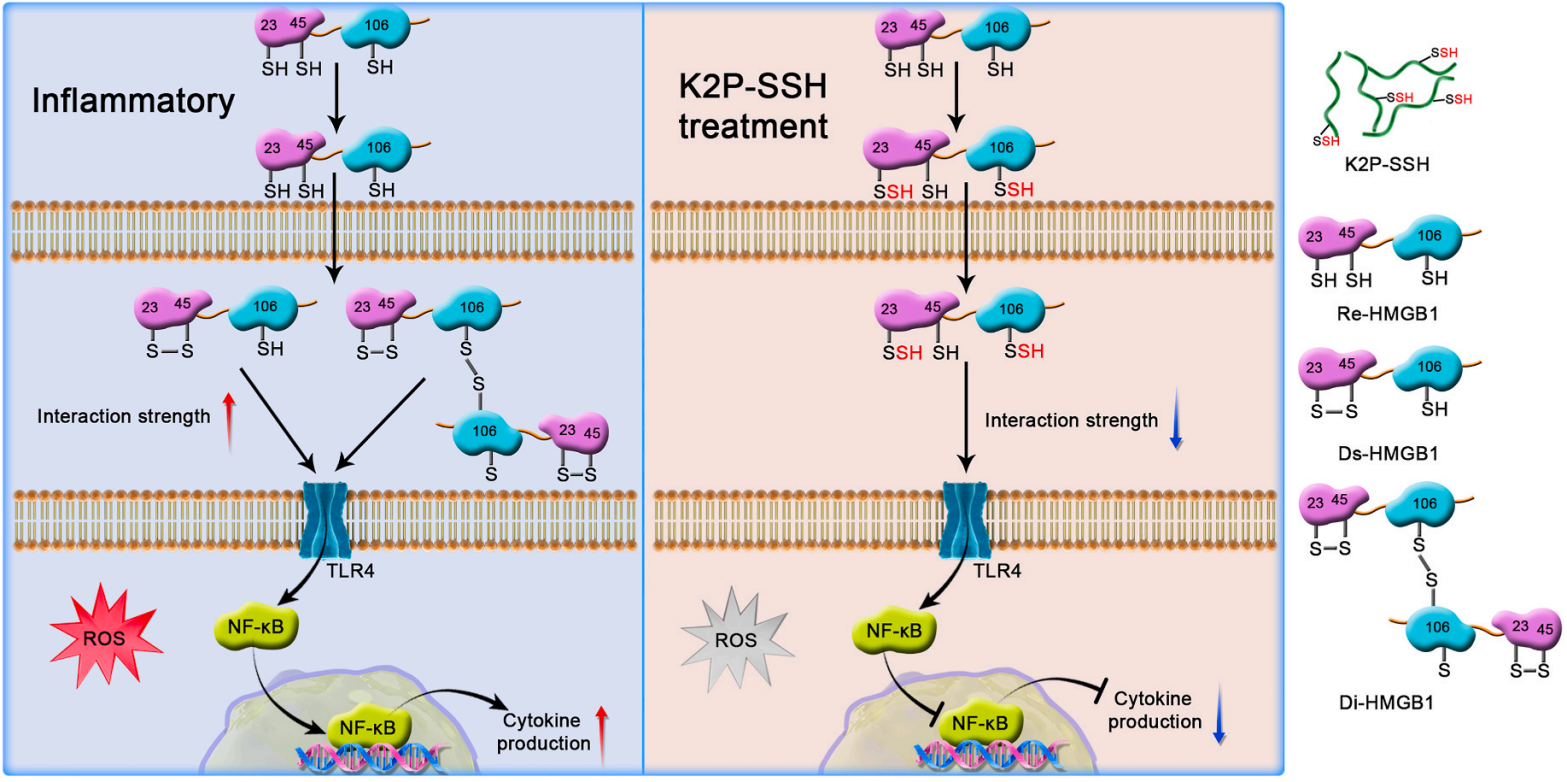
**Schematic depiction of the protective mechanisms of K2P-SSH against inflammatory and oxidative stress.** Under inflammatory conditions, HMGB1 undergoes oxidation and disulfide bond formation at Cys23, Cys45 and Cys106, promoting HMGB1 dimerization and HMGB1–TLR4 interaction, thereby activating NF-κB signaling and pro-inflammatory cytokine production. K2P-SSH promotes sulfhydration of HMGB1 at Cys23 and Cys106, which is associated with reduced disulfide bond formation and dimerization of HMGB1, thereby attenuating HMGB1–TLR4 interaction and downstream inflammatory signaling.

This study revealed that Cys23 and Cys106 of HMGB1 can be sulfhydrated by K2P-SSH. To investigate the potential effects of sulfhydration at Cys23 and Cys106 of HMGB1 on TLR4 interaction, structural docking analysis was performed to simulate conformational changes following HMGB1 sulfhydration. Compared with disulfide HMGB1 and dimerized HMGB1, sulfhydrated HMGB1 exhibited altered interaction patterns with the TLR4/MD-2 complex. The corresponding ZDOCK scores were 1427, 1465, and 1422 for disulfide, dimerized, and sulfhydrated HMGB1, respectively, suggesting that sulfhydration may influence HMGB1–TLR4/MD-2 recognition (Fig. 8A). To further assess HMGB1–TLR4/MD2 binding, SPR analysis was performed using wild-type HMGB1 and the HMGB1^C23SC106S^ double mutant. The results demonstrated that the HMGB1^C23SC106S^ mutant exhibited lower binding affinity toward TLR4/MD2 than wild-type HMGB1, with KD values of 74.1 μM and 5.24 μM, respectively (Fig. 8B and C). These findings support the importance of Cys23 and Cys106 in mediating HMGB1–TLR4/MD2 binding. We further evaluated HMGB1–TLR4 interaction by CO-IP assays. In HMGB1^WT^ cells, K2P-SSH treatment tended to reduce the normalized amount of TLR4 co-immunoprecipitated with Flag-HMGB1 compared with LPS-treated cells. In contrast, no comparable reduction in the normalized amount of TLR4 co-immunoprecipitated with Flag-HMGB1 was observed in HMGB1^C23SC106S^ cells following K2P-SSH treatment relative to LPS stimulation (Fig. 8D). Following a previously described method [20], we found that co-incubation of HMGB1 with LPS and H_2_O_2_ in serum promoted HMGB1 dimerization, whereas K2P-SSH tended to attenuate this process (Fig. 8E). Moreover, to evaluate the effects of K2P-SSH on disulfide and dimerized HMGB1 *in vivo*, we examined HMGB1 in mouse serum by Western blot. Following intraperitoneal injection of LPS, both disulfide and dimerized HMGB1 were detected in serum. Oral administration of K2 significantly reduced disulfide HMGB1 levels and showed a trend toward lower levels of dimerized HMGB1 (Fig. 8F). To further evaluate the role of Cys23 and Cys106 in HMGB1-mediated inflammatory signaling, inflammatory cytokine release was examined in cells expressing wild-type HMGB1 or the HMGB1^C23SC106S^ mutant. LPS stimulation increased TNF-α, IL-6, and IL-1β release in wild-type HMGB1 cells, whereas K2P-SSH treatment showed a trend toward reduced cytokine production. In contrast, cytokine induction was less pronounced in HMGB1^C23SC106S^ mutant cells, and the inhibitory effects of K2P-SSH were attenuated (Fig. 8G). Collectively, these findings suggest that K2P-SSH-mediated sulfhydration interferes with disulfide bond formation at Cys23 and Cys106 of HMGB1, thereby reducing the generation of disulfide and dimerized HMGB1 and attenuating HMGB1–TLR4 interaction and downstream inflammatory signaling (Fig. 9).

## 4 Discussion

This study screened and identified keratin K2 as a protein with potent anti-inflammatory and antioxidant properties. Notably, K2 is widely distributed—albeit at varying levels—in keratinous animal tissues, including goat horn, yak horn, rhinoceros horn, and saiga antelope horn. In traditional Asian medicine, these materials have been used for over a thousand years to exert antipyretic and antihypertensive effects [1,52], which are likely attributable to keratins such as K2. Mechanistically, our findings suggest that K2 converts H_2_S into protective sulfhydration signals, including HMGB1 sulfhydration, which contributes to its anti-inflammatory effects.

Widespread and reversible sulfhydration constitutes a dynamic oxidative defense system capable of counteracting systemic redox imbalances. The protective mechanisms of sulfhydration reported primarily involve: direct scavenging of ROS through electron donation by -SSH groups [45]; and restoration of oxidized protein thiols to their reduced state, thereby functioning as a “rescue loop” that prevents excessive oxidative damage [10,46]. Furthermore, sulfhydration of specific proteins serves as a “molecular switch” that modulates their activity, consequently exerting anti-inflammatory and antioxidant effects [[53], [54], [55], [56]].

The gastrointestinal tract is one of the major sites of endogenous sulfur metabolism, where gut microbiota continuously generates H_2_S and RSS. Recent studies have demonstrated that microbiota-derived RSS contribute substantially to host antioxidant defense, whereas impaired intestinal sulfide metabolism results in excessive H_2_S accumulation, mitochondrial dysfunction, and systemic inflammatory responses [3,41]. In agreement with these observations, our findings indicate that K2 alleviates inflammation by maintaining H_2_S homeostasis rather than simply scavenging H_2_S. By utilizing excessive H_2_S to generate protective sulfhydration signals, K2 promotes sulfhydration of key inflammatory regulators, including HMGB1, p65, and Keap1, thereby linking gut sulfur metabolism with systemic redox regulation.

The HMGB1/TLR4/NF-κB signaling pathway is central to oxidative-inflammatory stress. HMGB1 binding to TLR4 is primarily mediated by its B box domain (amino acids 89–162) interacting with MD-2, the co-receptor component of the TLR4/MD-2 complex [57]. The redox states of Cys23, Cys45, and Cys106 critically determine HMGB1's ability to bind TLR4, with disulfide and dimerized HMGB1 representing the two primary binding-competent forms [50]. Our results suggest that K2P-SSH-induced sulfhydration of HMGB1 is associated with alterations in its binding-competent conformation and reduced HMGB1–TLR4 interaction.

In this study, we found that K2P-SSH-mediated sulfhydration of p65 and Keap1 was associated with anti-inflammatory and antioxidant effects, accompanied by reduced p65 nuclear translocation and enhanced Nrf2 nuclear accumulation. Previous studies have shown that p65 Cys38 and Cys120 are located within the N-terminal Rel homology domain, which contains the DNA-binding interface of p65 [[58], [59], [60], [61]]. Sulfhydration at p65 C120 may inhibit p65–DNA binding, thus suppressing NF-κB transcriptional activity. Under oxidative stress, intracellular Keap1 forms disulfide bonds between Cys613 and Cys 226, as well as among Cys 622/Cys 624, leading to its dissociation from Nrf2 and subsequent Nrf2 nuclear translocation, which activates antioxidant defense pathways [62]. Sulfhydration at Keap1 C613 may interfere with the formation of these disulfide bonds, thereby modulating Keap1–Nrf2 dissociation and downstream antioxidant responses. Our results demonstrate that K2P-SSH sulfhydrates p65 at Cys120 and Keap1 at Cys613, consequently reducing p65 nuclear translocation while promoting Nrf2 nuclear accumulation to exert anti-inflammatory and antioxidant effects. The underlying mechanisms, however, warrant further investigation.

Previous studies have mainly focused on H_2_S donors such as NaHS and GYY4137 as inducers of protein sulfhydration [6]. In contrast, our study demonstrates that a naturally derived keratin protein can utilize endogenous H_2_S to promote protein sulfhydration without directly serving as a conventional H_2_S donor. This finding expands the current understanding of sulfhydration regulation and suggests an alternative strategy for modulating H_2_S signaling.

Using K2 as an example, this work investigates the *in vivo* behavior of keratin, including its regulation of H_2_S homeostasis, the targeting of specific proteins, and the underlying mechanism of action. Several potential mechanisms may contribute to the therapeutic effects of K2. First, given the inherent antioxidant activity of RSS [63], K2 may exert its effects by modulating endogenous RSS levels. Second, sulfhydration of transsulfuration pathway proteins such as S-adenosyl homocysteine hydrolase (SAHH) and methionine adenosyltransferase 2 A (MAT2A) can inhibit amino acid metabolism and induce ferroptosis [6]; K2 may regulate the redox status of these proteins, influencing amino acid metabolism to exert its effects. Third, sulfhydration of intestinal receptors such as transient receptor potential vanilloid 1 (TRPV1) and metabotropic glutamate receptor 5 (mGluR 5) has been implicated in gut-related signaling [42,64]; K2 may modulate these proteins to transmit neural signals, contributing to its rapid antipyretic effects. These avenues merit deeper exploration.

This study also has several limitations. Although RCCP is an effective strategy for enriching sulfhydrated proteins, it may co-enrich proteins indirectly linked through disulfide interactions. Therefore, RCCP-identified proteins should be considered putatively sulfhydrated proteins unless further validated. In this study, key targets, including HMGB1, p65, and Keap1, were further confirmed by QTRP analysis, site-directed mutagenesis, and functional assays. However, additional RCCP-identified candidates will require further validation, and direct *in vivo* evidence linking site-specific sulfhydration to anti-inflammatory and antioxidant effects remains limited. Moreover, the upstream regulation of H_2_S metabolism and the contribution of gut microbiota-derived sulfur metabolites to K2-mediated sulfide regulation warrant further investigation. Finally, the therapeutic potential of K2 requires validation in additional disease models and clinical samples.

Collectively, these findings provide a mechanistic basis for developing K2-based biomimetic therapeutics with potential applications in human health. By integrating interdisciplinary approaches—such as genetic engineering, synthetic biology, and artificial intelligence—we aim to design recombinant proteins and peptides that replicate the therapeutic benefits of endangered animal-derived materials, such as rhinoceros horn and saiga antelope horn. This strategy offers a viable substitute for rare animal-based medicinal substances, contributing to wildlife conservation and supporting the goal of harmonious coexistence between humans and nature [65].

## 5 Conclusion

Overall, our study identified K2 as a hyperreactive thiol-containing protein commonly present in keratinous medicinal materials. K2 regulates H_2_S homeostasis and promotes protein sulfhydration, thereby modulating protein redox status and cellular stress responses. Specifically, under oxidative-inflammatory stress, K2 is converted *in vivo* to K2P-SSH following reaction with H_2_S. K2P-SSH promoted sulfhydration of HMGB1 at Cys23 and Cys106, accompanied by modulation of the Nrf2/Keap1 and NF-κB signaling pathways. Collectively, as a key active constituent of keratin underlying its therapeutic effects, K2 represents a promising candidate for future drug development and provides a scientific rationale for wildlife conservation.

## 6 Declaration of generative AI and AI-assisted technologies in the manuscript preparation process

During the preparation of this manuscript, the authors used ChatGPT solely for language translation. The tool was not used for the generation or interpretation of scientific content. After using this tool, the authors carefully reviewed and edited the manuscript as necessary and take full responsibility for the content of the published article.

## CRediT authorship contribution statement

**Xiaozheng Huang:** Supervision, Writing – review & editing. **Wanglin Bao:** Data curation, Investigation, Writing – original draft. **Shengming Tang:** Data curation, Investigation, Writing – original draft. **Miao Li:** Formal analysis. **Yuqing Sun:** Formal analysis. **Hui Ge:** Formal analysis. **Yuxin Zhao:** Methodology. **Hao Yuan:** Methodology. **Xinyu Pan:** Methodology. **Wei Lin:** Project administration. **Yuanqing Wei:** Project administration. **Shuying Han:** Project administration. **Wenxing Wu:** Conceptualization, Supervision, Writing – review & editing. **Rui Liu:** Conceptualization, Supervision, Writing – review & editing. **Jin-ao Duan:** Conceptualization, Supervision, Writing – review & editing.

## 7 Ethics declaration

This study was conducted in accordance with the ARRIVE (Animal Research: Reporting of In Vivo Experiments) guidelines. This study was approved by the Institutional Animal Care and Use Committee of Nanjing University of Chinese Medicine. (Approval No. #202408A041, #202410A029, #202501A062)

## Declaration of competing interest

The authors declare that they have no known competing financial interests or personal relationships that could have appeared to influence the work reported in this paper.

## Data Availability

Data will be made available on request.
